# Analogs of Precambrian microbial communities formed *de novo* in Caucasian mineral water aquifers

**DOI:** 10.1128/mbio.02831-24

**Published:** 2024-12-11

**Authors:** Daria G. Zavarzina, Alexey A. Maslov, Alexander Y. Merkel, Nataliya A. Kharitonova, Alexandra A. Klyukina, Ekaterina I. Baranovskaya, Elena A. Baydariko, Evgeniy G. Potapov, Kseniya S. Zayulina, Andrey Y. Bychkov, Nikolay A. Chernyh, Elizaveta A. Bonch-Osmolovskaya, Sergey N. Gavrilov

**Affiliations:** 1 Winogradsky Institute of Microbiology, Research Centre of Biotechnology, Russian Academy of Sciences, Moscow, Russia; 2 Department of Geology, Lomonosov Moscow State University, Moscow, Russia; 3 Pyatigorsk Research Institute of Balneology, North Caucasus Federal Scientific and Clinical Center, Pyatigorsk, Stavropolʹskiy kray, Russia; 4 Department of Biology, Lomonosov Moscow State University, Moscow, Russia; Georgia Institute of Technology, Atlanta, Georgia, USA

**Keywords:** mineral water basins, aquifers, continental subsurface biosphere, anaerobic microbial community, Last Universal Common Ancestor

## Abstract

The microbiome of deep continental aquifers is considered the most slowly evolving part of the biosphere. The Yessentukskoye Mineral Water Basin (YMWB), located in the pre-Caucasus region, contains three closely spaced but distinct aquifers, the Upper Cretaceous, the Lower Cretaceous, and the Upper Jurassic, which represent unique objects for subsurface biosphere research due to gas-hydrogeochemical and thermal anomalies of the area. We analyzed the geological and hydrogeochemical parameters of the three aquifers and a recharge area of the YMWB and investigated their microbial communities using metagenomic and cultivation-based approaches within a long-term survey. Correlation analysis of the obtained data revealed stable and highly stratified microbial communities inhabiting four distinct ecosystems. Their structure and the metabolic traits of their prokaryotic populations were similar to those presumed to have dominated the Earth’s biosphere during several critical periods of its evolutionary history, that is, the Early Archean, the period of banded iron formations accumulation, and the Great Oxidation Event. Among the YMWB strata, the Upper Jurassic aquifer, supersaturated with CO_2_, influenced by magmatic activity, and highly enriched with thermophilic autotrophic hydrogenotrophic acetogens, turned out to be the first described modern ecosystem based on the primary production by a process predicted to support the Last Universal Common Ancestor (LUCA). The characterization of the YMWB microbial communities reveals a contemporary model environment of the early stages of Earth’s development and thus contributes to the understanding of the evolutionary traits in microbial populations that may have played a critical role in the formation of the modern biosphere.

**IMPORTANCE:**

Continental subsurface environments are estimated to harbor up to one-fifth of the planet’s total biomass, representing the most stable and slowly evolving part of the biosphere. Among the deep subsurface inhabitants, the microbial communities of drinking mineral waters remain the least studied. Our interdisciplinary study of the Yessentukskoye Mineral Water Basin shows how hydrochemical and hydrodynamic factors shape different subsurface ecosystems, whose microbial populations influence the composition of mineral waters. A comprehensive analysis reveals the similarity of these ecosystems to those predicted for the early Earth. The deepest of the studied aquifers is the first described modern ecosystem with the most probable primary producer performing hydrogenotrophic acetogenesis. Thus, our results contribute to the understanding of the genesis of modern drinking water resources and expand the knowledge of the evolutionary traits that may have played a critical role in the formation of the Earth’s biosphere.

## INTRODUCTION

Subsurface environments still hold many scientific mysteries due to an incredible diversity of spatially separated geochemical settings. The deep continental biosphere (DCB), which is currently estimated to contain up to 19% of the planet’s total biomass (1–4), is considered to be the most stable and slowly evolving part of the biosphere (5). Microorganisms in DCB develop in the absence of light, in the presence of mineral energy sources, and with limited nutrient diffusion due to water spreading through cavities in the rocks (6–8). Deep subsurface environments are characterized by low rates of mass transfer, which determine the low rates of metabolic processes in microorganisms of the DCB down to 10^−4^ to 10^−3^ fmol of an electron acceptor cell^−1^ d^−1^ (5). Given the relative stability of the composition of igneous and sedimentary rocks throughout Earth’s geological history and the predominance of anaerobic conditions in the DCB, specific microbiological processes occurring in the modern subsurface can be considered a contemporary model of the early stages of biosphere development. Among the DCB ecosystems, deep aquifers are not overly enriched in organic matter, as seen in oil and gas reservoirs, or in metals, as found in deep ore deposits of iron, sulfides, polymetals, uranium, and gold, such as the deepest South African mines (9), and they represent a more appropriate model of early Earth’s biocenoses. However, subsurface water microbiomes unrelated to oil or ore deposits have been much less studied so far (10–12).

The Yessentukskoye mineral water basin (YMWB) makes a unique object within the framework of DCB research. Despite the relative geological youth of its sedimentary cover, it has many common features with hypothetical ecosystems of an ancient Earth, such as the East Pilbara Granite–Greenstone Terrane, which is part of Pilbara craton deposited between 3515 and 3320 Ma by repeated cycles of plume-related mafic–felsic volcanism onto 3725–3600 Ma continental crust (13, 14). Peculiar features of YMWB include the small thickness of sedimentary deposits, the proximity of magma sources, the presence of tectonic faults and intrusions formed during the Alpine folding, and, finally, a steady flow of gas and water fluids actively interacting with sedimentary rocks. The complex geological structure strongly influences the chemical composition of the water and forms several distinct but closely located aquifers within the Upper Jurassic (UJ, Titonian-Valanginian, J_3_*tt*-K_1_*v*), Lower Cretaceous (LC, Aptian-Lower Albian, K_1_*a-al*_1_), and Upper Cretaceous (UC, Cenomanian-Maastrichtian, K_2_*s-m*) sedimentary rocks (15–18). The main feature of the YMWB is a gas-hydrogeochemical and thermal anomaly (inversion) of mineral waters. Slightly oxygenated warm freshwater of the LC aquifer is located between the underlying UJ aquifer containing thermal carbonaceous waters supersaturated with CO_2_ and the overlying UC aquifer containing warm freshwater in its recharge area (RA) and thermal carbonaceous waters with free gas phase in its deep part.

Our preliminary studies revealed significantly different phylogenetic profiles of prokaryotic populations in two wells of the UC aquifer, highlighting the predominance of uncultured *Hadarchaeota* and *Actinomycetota* in them (19). These wells extract the most balneologically valuable water types of YMWB (brand “Essentuki” type 4 and type 17 waters). They differ from each other in mineralization and are commercially extracted from the beginning of the 19th century. Their main therapeutic properties are provided by the high concentration of sodium (bi)carbonate and boron (18, 20, 21). Here, we present the results of a comprehensive characterization of YMWB subsurface ecosystems in terms of their geology, assessed through a retrospective analysis of archived data, as well as their geochemistry and phylogenetic and metabolic diversity of their microbial communities. We also discuss the interconnections between the physicochemical parameters of the ecosystems and the metabolic capacities of microbial communities inhabiting them. Among the communities studied there, one is of special interest, as it is dominated by a hydrogenotrophic autotrophic acetogenic bacterium which represents 80%–95% of the community and may fuel the entire ecosystem.

## MATERIALS AND METHODS

### Characteristics of the studied wells and sampling spots

Prior to water sampling for microbiological investigations, the studied wells were operated at full load for 18 months. Flow data as a function of well volumes per time unit (Table S1) testify that microorganisms that may have grown in the wellbore rather than in the sampled aquifer were definitely washed out by the extraction of hundreds to thousands of well volumes before sampling began. Such a prolonged maximum operational load of the wells also minimized the bias of sample contamination and unequal recovery of immobilized and planktonic cells from the subsurface. The water produced by the YMWB is used for medical drinking. Therefore, the well design is of the highest standard in contamination prevention (Fig. S1). During our study, we sampled three different subsurface aquifers through five wells (Table S1). We also used an additional well 70, drilled in the recharge area of the UC aquifer, as a reference spot.

### Chemical analyses of mineral water and gas phase samples

All samples for hydrochemical analysis were prepared as described previously (20, 21). Major anions (F^-^, Cl^-^, Br^-^, SO_4_^2-^, and NO_3_^-^) and cations (Ca^2+^, Mg^2+^, Na^+^, and K^+^) were analyzed by liquid ion chromatography (Shimadzu LC-20, Japan). The trace elements were determined using the inductively coupled plasma mass spectrometry (ICP-MS) technique on a high-resolution mass spectrometer with inductively coupled plasma ionization element-2 (Thermo, Germany). Total organic carbon (TOC) was determined in water samples using an infrared detector on a Shimadzu TOC analyzer (TOC-V). Samples of spontaneous gases were collected in glass tubes by the replacement method (21). The concentration of components (He, H_2_, N_2_, СН_4_, and O_2_) in gas samples was determined using a gas chromatograph Crystall 5000.2 (Chromatek, Russia) using thermal conductivity detectors and molecular sieve columns, with He and Ar as carrier gases. Detection limit for H_2_ was 10 ppm.

### Stable isotope composition of water

Samples were analyzed for stable isotopes (δ^2^H_(H2O)_, δ^18^O_(H2O)_) using Finnigan MAT253 mass spectrometer (Thermo Finnigan, Germany) operating in a continuous He-flow mode, after on-line pyrolysis with a Thermo Finnigan high temperature conversion elemental analyzer (TC/EA). The results were retrieved in standard delta notation relative to Standard Mean Ocean Water (SMOW).^13^C/^12^C isotope ratio of total dissolved inorganic carbon (TDIC) expressed as δ^13^C_TDIC_ ‰ VPDB was analyzed by mass spectrometry on a Delta V Advantage plus Gas Bench II mass spectrometer (Thermo Finnigan, Germany) according to the procedure described by Salata et al. (22). The ^13^C/^12^C isotope ratios in CO_2_ and CH_4_ (expressed as δ^13^C in ‰ relative to the PDB International Standard) were determined using the same mass spectrometry technique. The analytical error was less than 0.25‰.

### Microbial community characterization

#### Sampling

For DNA isolation, mineral waters were sampled directly from production wells using a pre-sterilized membrane filtration unit FM02-1000 with a sterile 0.2 µm pore size track membrane filter (Fig. S1), and 100 L of water was filtered for each sample as described previously (19, 23).

Water samples serving as the inocula and media for primary enrichments were taken directly from the wellheads with sterile 10 mL syringes and needles and were transferred immediately after sampling to sterile 17 mL Hungate anaerobic culture tubes prefilled with N_2_/CO_2_ gas mixture (80/20 vol/vol). In the case of gas-metabolizing microorganisms, 5 mL water samples was added; in other cases, water sample volumes comprised 10 mL.

In total, three samples from each of the six production wells were taken and analyzed; 16S rRNA and metagenomic sequencing for each of the 18 samples was performed in triplicates.

### DNA isolation, preparation, sequencing, and analysis of 16S rRNA gene amplicon libraries

DNA was isolated directly from fixed membrane filters as previously described (19) using the FastDNA SPIN Kit for Soil (MP Biomedicals, USA) according to the manufacturer’s instructions. Amplicon libraries of the V4 region of the 16S rRNA gene were prepared, sequenced, and analyzed using Illumina technology as previously described (24) and universal prokaryotic primer system 515F (5′-GTGBCAGCMGCCGCGGTAA-3′) (25) and Pro-mod-805R (5′-GACTACNVGGGTMTCTAATCC-3′) (26). The libraries were prepared and sequenced in two replicates for each sample. The amplicon sequence variant (ASV) table was constructed using the Dada2 script (27) and the SILVA 138.1 database (28). Analysis of the ASV table was performed using MicrobiomeAnalyst (29).

### Preparation, sequencing, and analysis of metagenomic libraries

A shotgun metagenome library preparation and sequencing were performed commercially by BioSpark Ltd., Moscow, Russia, using the KAPA HyperPlus Library Preparation Kit (KAPA Biosystems, UK) according to the manufacturer’s protocol and NovaSeq 6000 system (Illumina, San Diego, CA, USA) with the reagent kit, which can read 100 nucleotides from each end. Raw reads were processed with Cutadapt (30) and Trimmomatic (31) for adapter removal and quality filtering. Reads were processed in MetaWRAP (32) using MetaSPAdes (33) and MEGAHIT (34) for assembly, MaxBin 2 (35), MetaBAT 2 (36), and CONCOCT (37) for binning, and Salmon (38) for coverage calculation. Bin completeness and contamination were evaluated using CheckM (39). Taxonomies were assigned to each bin using GTDBtk (40). The characteristics of the resulting MAGs are given in Tables S3 and S4. The resulting MAGs data set was screened for the presence of key genes that determine basic energy conservation and biomass production processes expected to be favorable for prokaryotes in the determined geochemical settings. The pathways were first screened for using the corresponding Kyoto Encyclopedia of Genes and Genomes (KEGG) modules or custom gene sets determining a metabolic feature according to previously published data. For each of the metabolic features, several alternative KEGG modules or gene sets were screened in the MAGs, which passed the quality check. Key proteins and genes of the modules and custom gene sets are listed in Table S5. Completeness of the metabolic modules or gene sets was examined prior to assigning a metabolic capability to a genotype (the range of completeness to be considered in each case is provided in Table S5). After passing the completeness check, each set of identified genes was examined to encode the proteins that are necessary and sufficient to reliably assign a metabolic feature to the organism. These proteins were chosen based on a comprehensive scientific publications’ analysis as of August 2024. The selected key proteins and the cutoffs for the identification of their homologs in MAGs are given in Table S5. The following peculiarities in screening for the target metabolic features should be mentioned. Detailed lists of enzymes used to determine carbon fixation pathways in the MAGs are provided in Fig. S5. KEGG modules for the reductive glycine pathway and reverse oxidative TCA (roTCA) cycle are absent in public databases; the necessary gene IDs were retrieved from (41) and (42). The description of the “CO to acetate pathway” and the genes necessary for its identification were taken from (43). A low identity percentage cutoff (20%–30%) for CO-dehydrogenases and multiheme cytochromes was applied, considering the wide phylogenetic diversity of these groups of enzymes as discussed in (44) and (45). Query sequences for the identification were retrieved from different public databases according to the IDs found in the corresponding publications that are listed in Table S5.

### Enrichment procedure

The filled Hungate tubes were supplied with energy substrates and electron acceptors and incubated in the dark at the average temperature observed during the well operation in tap mode (Table 1). The target metabolic groups of microorganisms were as follows: organo/lithotrophic sulfate reducers; organo/lithotrophic iron reducers; anaerobic iron oxidizers; organo/lithotrophic methanogens; organotrophic Archaea (cultivated with bacteriocins); syntrophic acetate oxidizers; and fermentative organotrophs. The substrate combinations tested are summarized in Table 1. Sodium sulfide (0.5 g L^−1^) was added as a reducing agent in all cases, except those aimed at the enrichment of iron reducers and oxidizers. The latter group was enriched in the presence of selected grains of siderite FeCO_3_ (Bakal deposit, Urals, Russia) with the purity confirmed by X-ray diffraction. The grains were grounded to powder (<100 µm particle size) and added to Hungate tubes prior to sterilization. Synthesized ferrihydrite for iron reducers was prepared as previously described (46). In case of positive growth, 5 mL of the enrichment was used for DNA extraction and 16S rRNA gene profiling, whereas the remaining volume was used for the next transfer (5% vol/vol) to a fresh medium containing the target substrate (Table 1). Basic mineral composition of the media was as follows (g L^−1^): NH_4_Cl, 0.33; KCl, 0.33; KH_2_PO_4_, 0.33; CaCl_2_ ×2H_2_O, 0.33; MgCl_2_ ×6H_2_O, 0.33; NaCl, 1.0 (for enrichments from wells 49-E, 71, 75-bis) or 2.0 (well 46); NaHCO_3_, 6.0 (wells 49-E, 71, 75-bis) or 9.0 (well 46); Na_2_S × 9H_2_O, 0.5 (if a reductant was necessary); 1 mL L^−1^ trace element solution, and 1 mL L^−1^ vitamin solution (23).

**TABLE 1:** Key components of the media used to obtain primary enrichment cultures of target metabolic groups

Target metabolic group	Key components (concentration)			
	Electron acceptor	Electron donor	Carbon source	Bacteriocins
Organotrophic sulfate reducers	SO_4_^2-^ (20 мМ)	Lactate (10 мМ)	Acetate (2 мМ)	na^*a*^
Lithotrophic sulfate reducers	SO_4_^2-^ (20 мМ)	Formate (10 мМ)	Acetate (2 мМ)	na
Organotrophic iron reducers	SF^*b*^ (Fe^3+^, 10 мМ)	Acetate (10 мМ)		na
Lithotrophic iron reducers	SF (Fe^3+^, 10 мМ)	Formate (10 мМ)		na
Anaerobic iron oxidizers	FeCO_3_ (172 mM)	СО_2_ (20% vol/vol)	Acetate (2 мМ)	na
Lithotrophic methanogens	СО_2_ (20% vol/vol)	Formate (10 мМ)	СО_2_ (20% vol/vol)	Ampicillin (2.9 мМ)
Hydrogenotrophic methanogens	СО_2_ (20% vol/vol)	H_2_ (50% vol/vol)	СО_2_ (20% vol/vol)	Ampicillin (2.9 мМ)
Organotrophic archaea	Yeast extract (1 g/L) + glucose (1 g/L)			Ampicillin (2.9 мМ)
Acetate oxidizers	Acetate (10 mM)		СО_2_ (20% vol/vol)	na
Organotrophs	Yeast extract (1 g/L) + glucose (1 g/L)			na

^*a*^ na, not added.

^*b*^ SF, synthesized ferrihydrite.

Methanogens were isolated into pure cultures by several successive transfers of primary enrichment cultures, in which methane formation was detected, with ampicillin (2.9 mM) added to the medium. Iron reducers were isolated into pure cultures on selective media by 10-fold dilution method as described previously (23).

Microbial growth was monitored by the phase contrast and fluorescence microscopy using an Axio Lab.A1 microscope (Zeiss, Germany). In the cultures with iron minerals, cells were stained with acridine orange. Methane was determined using a Crystall 5000.2 gas chromatograph (Chromatek, Russia). Sulfide production was determined according to (47); Fe(II) production was monitored with ferrozine (48), and Fe(II) minerals were dissolved with 6 N HCl prior to analysis.

### Calculation of estimated values

The temperature in the aquifers penetrated by the investigated wells was reliably estimated using a variety of geothermometers (49–55) (Table S6).

The pH values of the water inside the aquifers were calculated based on the following assumptions: the gas-water ratio was determined by a gas factor, and the pressure corresponded to the depth of the water intake interval. Temperatures were set according to calculations using two SiO_2_-geothermometers (Table S6). The thermodynamic properties of calcium ions and calcium and sodium carbonate complexes were taken from previously published data (56).

The optimal growth temperature was estimated for the organisms whose genomes were assembled from the metagenomic data. The temperature optima were calculated based on the amino acid composition of the proteins encoded in the MAGs according to (57).

## RESULTS

### Geological and hydrogeochemical settings of YMWB

**Fig 1:**
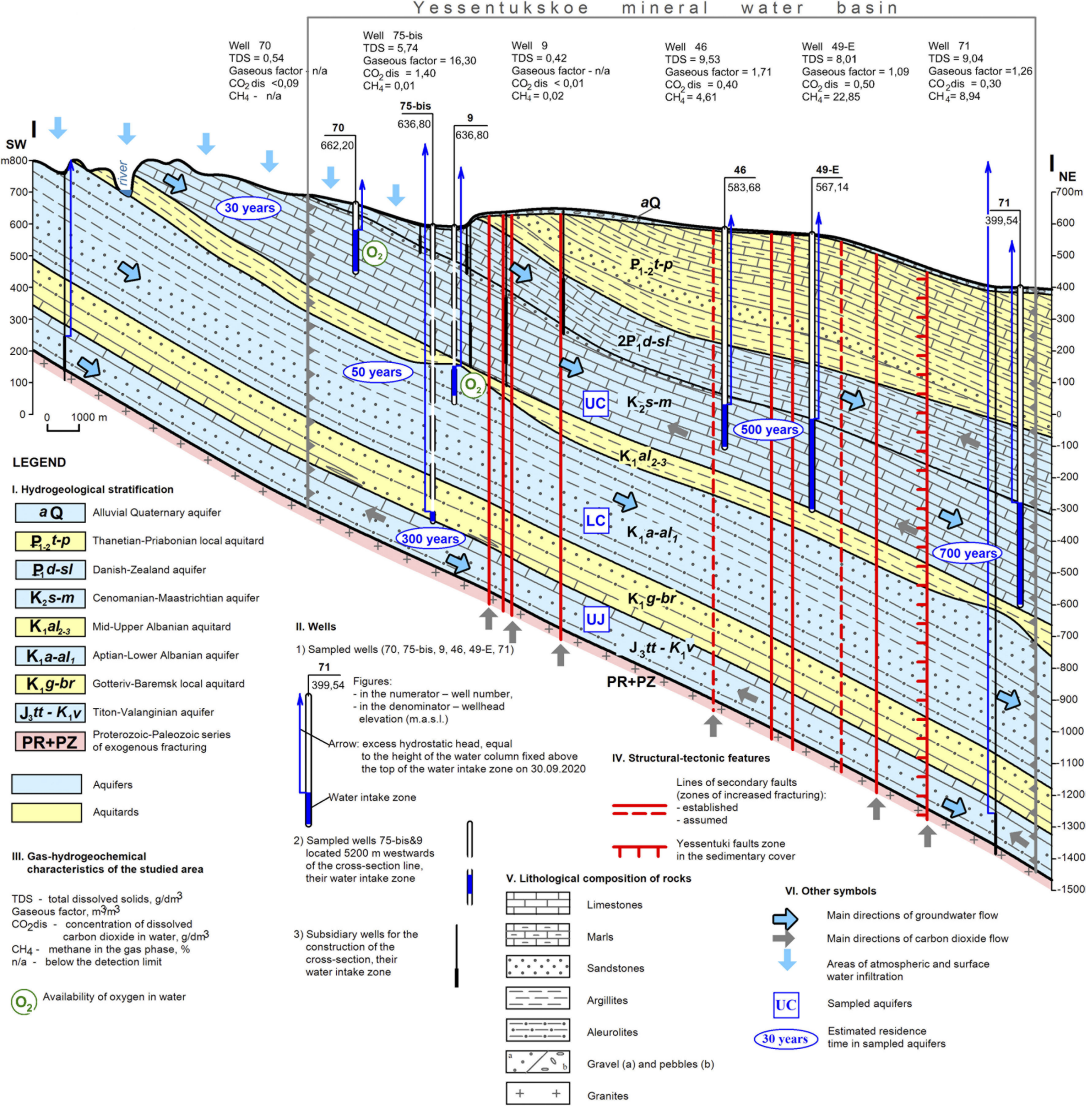
Hydrogeologic cross-section showing the location of the sampled wells and important hydrogeochemical and geological features of the YMWB. Aquifers are shown in blue, and aquitards are in yellow. The flow direction of water and dissolved gases is indicated by arrows.

To create a hydrogeological model of the basin, we compiled all the geological and hydrogeochemical information that was collected on the YMWB to date, including unpublished geological surveys of various years. The hydrogeological section of the area (Fig. 1; Fig. S2) is represented by monoclinal strata of four Meso-Cenozoic aquifers, separated by clay aquitards. The groundwater is characterized by a gradual vertical and lateral change in hydrogeological and hydrogeochemical parameters in a northeast direction along the decline of the sedimentary rocks and water movement away from the RA of the UC aquifer. (Fig. 1; Table 2).

**TABLE 2:** Hydrochemical and geological characteristics of the sampled aquifers and investigated wells and waters within the YMWB

Aquifer		К_2_*s-m*				К_1_*а-al_1_*	J_3_*tt*-K_1_*v*
Abbrevation		RA	UC			LC	UJ
Characteristics	Units	Wells					
		70	46	49-E	71	9	75-bis
Well depth	m	212	686	865	999	600	974
Transmissivity of the aquifer	m^2^/day	6	30	>20	<1	27	>10
Specific water flow rate	m^2^/day	0.1	0.02	0.04	0.0005	0.04	0.007
Residence time^*b*^	year	30	400	500	700	50	300
Water type		Na-HCO_3_	Na-HCO_3_-Cl	Na-HCO_3_-Cl	Na-HCO_3_-Cl	Na-Ca-HCO_3_-SO_4_	Na-Ca-HCO_3_-Cl-SO_4_
рН (calculated pH)	pH units	8.5 (8.3)	6.7 (6.2)	6.6 (6.6)	6.8	7.9 (7.6)	6.3 (5.5)
Т (calculated T_SiO2_)	°C	19.7 (15.0)	35.6 (67.1)	30.2 (69.8)	40.5 (74.0)	21.9 (23.9)	20.2 (58.3)
Total dissolved salts (TDS)_meas_	g/L	0.4	6.7	5.6	6.8	0.4	4.1
TDS_calc_	g/L	0.5	9.5	8	9	0.4	5.7
CO_2dis_	mg/L	< 0.01	0.4	0.5	0.3	< 0.01	1.4
Na^+^	-//-	147	2584	2256	2616	117	632
K^+^	-//-	2.6	38.3	23	37.4	2.8	38.4
Ca**^2+^**	-//-	6.3	72.8	28.8	30.9	38.9	385
Mg**^2+^**	-//-	1.3	50.8	22.7	25.5	6.8	177
HCO_3_^-^	-//-	363	4961	4056	4382	217	3812
SO**_4_^2-^**	-//-	47.2	<0.1	<0.1	<0.1	164	829
Cl**^-^**	-//-	20.1	1821	1616	1943	41.1	691
F^-^	-//-	0.7	<0.3	<0.3	<0.3	<0.3	<0.3
Br^-^	-//-	0.1	6	4.5	6.1	0.1	2.8
B	-//-	0.5	5.1	5.5	5	0.1	0.5
Li^+^	-//-	0.02	0.3	0.5	0.6	0.01	0.4
NO_3_^-^	-//-	0.1	<0.1	<0.1	<0.1	<0.1	<0.1
SiO_2_	-//-	12	40.8	43.1	46.7	15.3	34.2
Тotal carbon (TC)	ppb	67.4	983	864	884	44.7	448
Total organic carbon (TOC)	-//-	1.2	20.7	33	23.4	1.3	40
Ba	-//-	32.8	1163.5	1324.5	1654.4	27.4	4.5
Sr	-//-	252.6	3733.2	4052.6	5091.9	955.9	6669.5
Fe	-//-	12.1	17	8.8	10.4	2.4	5.2
Mn	-//-	1.6	0.3	0.5	2.4	10.7	4.3
Cs	-//-	0.01	1.3	1.2	1.4	0.02	13
Rb	-//-	0.5	19.8	13.7	17.1	0.9	56.7
Zn	-//-	4.8	1.5	2.8	3	2.7	6.3
Co	-//-	0.6	0.1	0.1	0.1	0.05	0.2
Ni	-//-	1.9	0.7	1.1	1.6	0.4	2.3
Cu	-//-	3.4	1.4	2.6	4.4	0.7	6.2
δ^18^O_VSMOW_	‰	−11.5	−11.3	−11.5	−11.3	−14.2	−11.8
δD_VSMOW_	-//-	−81.2	−79	−85.9	−81.4	−99.4	−83.4
δ^13^C_DIC_	-//-	−5.02	2.11	2.48	−2.41	−14.7	ND^*a*^
δ^18^O_DIC_	-//-	−12	−6.2	−6.2	−10.3	−14.9	ND
Gaseous factor (gas/water)	m^3^/m^3^	0	1.71	1.09	1.26	0	16.3
O_2_	%	ND	0.23	0.52	0.53	19.72^*c*^	0.05
N_2_	-//-	ND	18.24	23.82	24.51	78.94^*c*^	0.45
CH_4_	-//-	ND	3.12	11.85	8.92	0.02^*c*^	0.004
CO_2_	-//-	ND	77.19	62.32	66.01	0.55^*c*^	99.44
H_2_	-//-	ND	0.01	0.02	0.01	ND	ND

^*a*^ ND, not determined.

^*b*^ Residence time of groundwater from the sampling wells determined by ^3^H concentration (Timokhin et al. Report on assessment of the resource potential of mineral and fresh groundwater of the Mineralovodskiy artesian basin. - Inozemtsevo: Kavkazgidrogeologiya, 2006. - Rosgeolfond No. 487117 (in Russian)).

^*c*^ Dissolved.

The RA penetrated by well 70 belongs to the zone of unconfined aquifers and has a direct connection with the ground surface affected by modern climatic, geomorphological, geological, and related processes. The lower part of the UC aquifer is penetrated by wells 46, 49-E, and 71 and belongs to the zone of confined aquifers with a low water flow rate. The zone is located far from the RA and is connected with the basement by sub-vertical faults, providing lateral fluid inflow from a deep magmatic chamber of the Byk Mountain (Fig. 1). The LC aquifer penetrated by well 9 belongs to the active water exchange zone, which is located far from its recharge area. It is characterized by a high rock permeability, which contributes to an unexpectedly faster and much deeper penetration of meteoric water compared with the overlying UC aquifer (Table 2). The deepest UJ aquifer penetrated by well 75-bis is remote from its recharge area, lies directly on the basement, and is strongly influenced by its geochemistry; full water exchange has not yet occurred there.

### Water chemistry

UC aquifer, the main productive aquifer of the YMWB, due to its complex hydrodynamic conditions, carries several types of pressurized water with different mineralization but almost identical major ionic compositions (Table 2; Fig. S3). In the RA, this aquifer contains fresh, warm, oxidized, slightly alkaline waters (well 70) of Na-HCO_3_ type lacking free gas phase, whereas the deeper, main submerged part of this aquifer (wells 46, 49-E, 71) contains thermal, reduced, neutral Na-HCO_3_-Cl waters with a gas factor (gas/water ratio) of 1.09–1.71 m^3^/m^3^ (Table 2). The free gas phase of these waters consists mainly of CO_2_ with the addition of N_2_ and biogenic CH_4_ with δ^13^С СН_4_ of −60% to −61% (Table S7). It is characterized by the highest amount of total carbon species, predominantly inorganic, ranging from 0.86 to 0.98 g L^−1^, and by high concentrations of Br, B, Sr, Ba, Rb, and Si (Table 2), indicating a close interaction of these mineral waters with the Byk Mountain intrusion.

LC aquifer, due to its short residence time and direct connection with the ground surface (Fig. 1; Table 2), contains a low concentration of dissolved oxygen and fresh, warm, neutral, sulfaceous pressurized water lacking the free gas phase (Table 2). The groundwater here has a low mineralization (about 0.4 g L^−1^) and belongs to the Ca-Na-Cl-SO_4_-HCO_3_ water type. The content of total carbon species and trace elements, such as Br, B, Sr, Ba, Rb, and Si, is very low (Table 2).

The deepest UJ aquifer contains thermal, reduced, pressurized water of Na-Ca-HCO_3_-Cl-SO_4_ type with the highest gas factor of 16.3 m^3^/m^3^ and mineralization of up to 5.7 g L^−1^. The free gas phase of this water contains 99.4% CO_2_. The total carbon content of the water is high, 448.0 ppb, and inorganic forms of carbon prevail. Elevated content of Sr, Rb, and Cs here indicates water interaction with the basement and its leaching (Table 2). The well 75-bis is characterized by maximal discrepancies between measured and calculated pH and temperature values. According to the SiO_2_ geothermometers (49, 50) and thermodynamic calculations, the actual values in this part of the UJ aquifer were pH 5.5 and T + 58°C (Table 2; Table S6).

Stable isotopic composition of oxygen and hydrogen in our samples revealed that the mineral waters of the UC aquifer, whether from RA or from its submerged part, are similar to each other, as well as to the atmospheric and surface waters of the region (Fig. S4). Most of the data obtained for these mineral waters are plotted along the global line of meteoric waters (GLMW), which indicates their infiltrative genesis. The LC aquifer (well 9) differs from all other aquifers by a lighter oxygen and hydrogen isotopic content of water in it. However, it is also plotted along the GLMW (Fig. S4).

**Fig 2:**
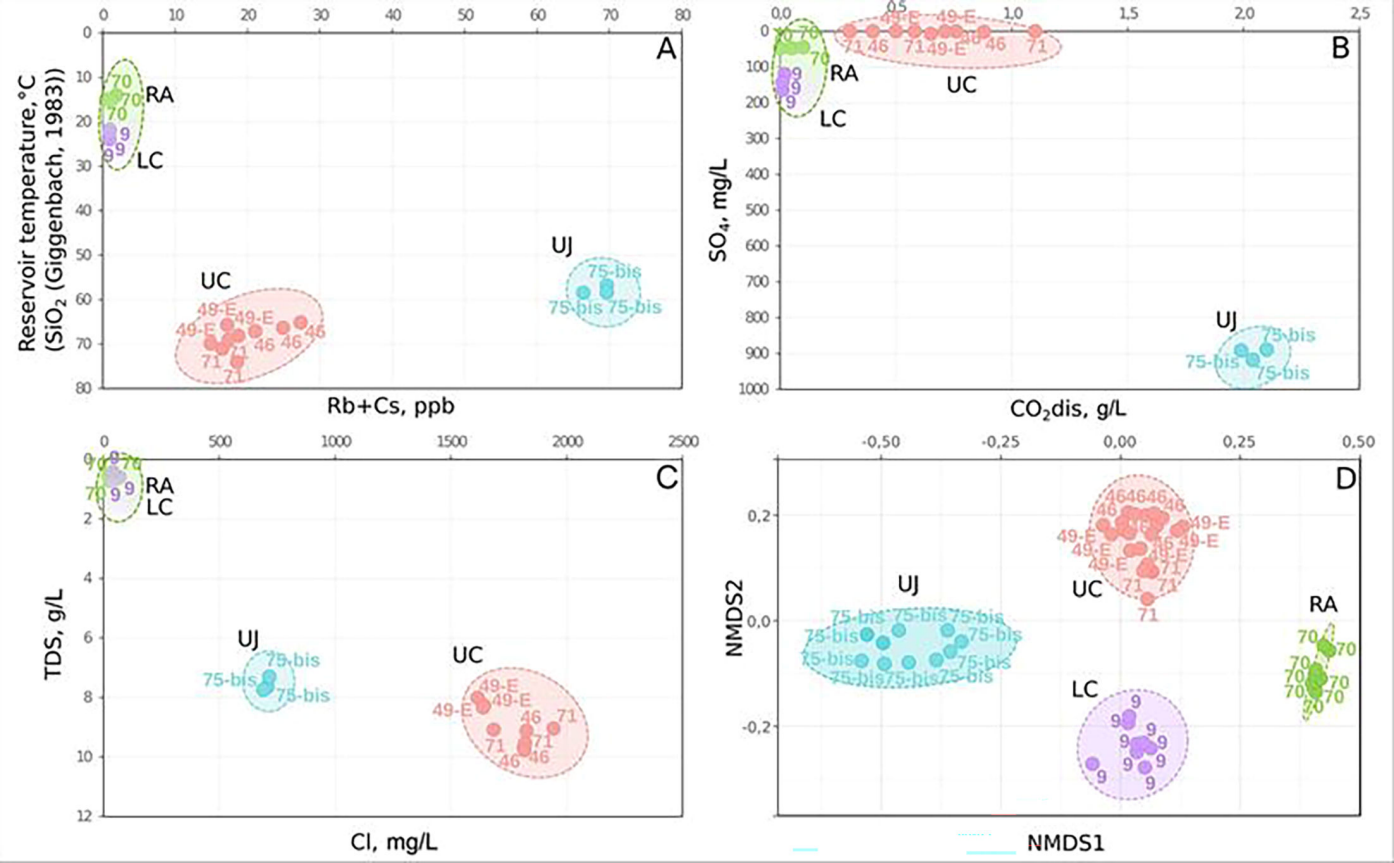
Grouping of wells by (**A**) the reservoir temperature, calculated using a SiO_2_ geothermometer (49, 50) and summarized Rb and Cs content (significance of differences assessed by PERMANOVA: F-value: 85.093; R-squared: 0.979; *P*-value: 0.001); (**B**) sulfate-ion and dissolved carbon dioxide content (significance of differences assessed by PERMANOVA: F-value: 4.383; R-squared: 0.706; *P*-value: 0.001); (**C**) TDS and chlorine-ion content (significance of differences assessed by PERMANOVA: F-value: 102.976; R-squared: 0.983; *P*-value: 0.001); and (**D**) beta-diversity calculations of corresponding microbial communities based on counting the difference in the representation of individual ASVs based on Jensen-Shannon divergence and non-metric multidimensional scaling (NMDS) ordination (significance of differences assessed by PERMANOVA: F-value: 29.821; R-squared: 0.68052; *P*-value: 0.001).

In terms of physico-chemical parameters indicating the microbial activity and characteristic features of different YMWB aquifers (calculated water temperature, Rb + Cs, SO_4_^2-^, and CO_2_, as the major indicators of microbial metabolic activity, Cl^-^ content and TDS demonstrated the hydrogeochemical inversion of YMWB mineral waters), the water samples extracted from the six wells could be divided into three substantially different groups (Fig. 2A through C): (i) warm, fresh, oxygenated water (wells 70 and 9); (ii); thermal, anaerobic, mineralized, gaseous, methane-containing water (wells 46, 49-E, 71); (iii) thermal, anaerobic, mineralized, supersaturated gaseous water with a high CO_2_ content (well 75-bis). The significance of sample clustering by geochemical parameters was supported by PERMANOVA tests (as detailed in Fig. 2 legend).

### Phylogenetic profiling of subsurface water samples

Beta-diversity calculations revealed a clear grouping of the analyzed microbial communities (Fig. 2D), which correlated with grouping of the physicochemical conditions of four different sampling areas of YMWB. The grouping was reproducible throughout all the sampling period. The significance of samples clustering by beta-diversity parameters was supported by PERMANOVA tests (as detailed in Fig. 2 legend).

**Fig 3:**
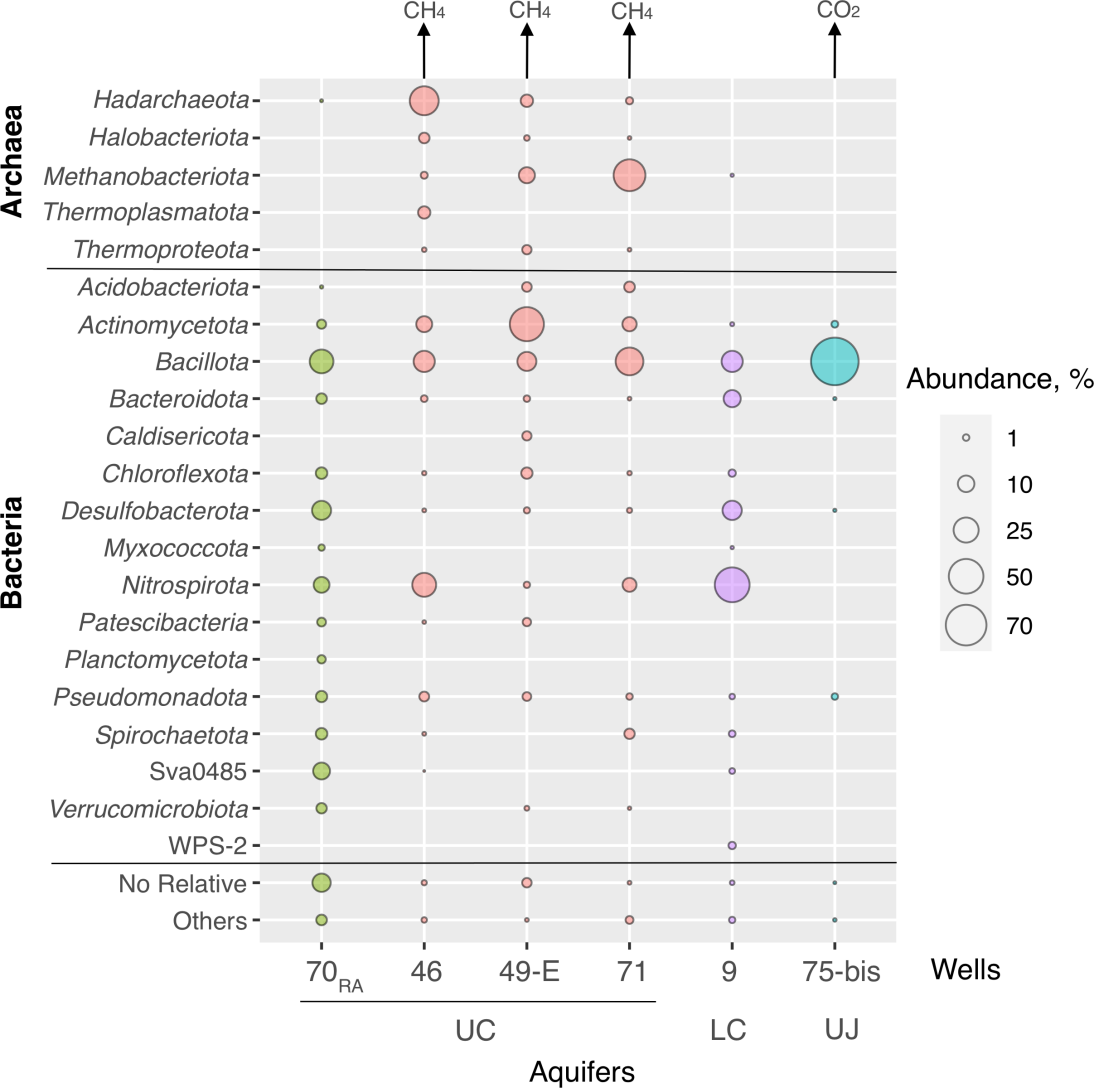
Phylogenetic diversity of YMWB microbial communities based on the results of high-throughput sequencing of 16S rRNA gene amplicons analyzed against the SILVA database, v.138. Phylum names are corrected according to references (58, 59).

Each well was sampled with different time intervals, at least three times over a period of 3 years (Table S1). 16S rRNA gene fragment profiling revealed diverse microbial communities in all the sampled wells. According to alpha-diversity calculations, the highest taxonomic diversity was observed for the community of the RA (well 70) and the lowest in the UJ aquifer (well 75-bis) (Table S8). The diversity of the communities decreased with depth and distance from the recharge area (Fig. 3; Tables S8 and S9). All the communities were predominated by uncultured groups of prokaryotes of novel genera to phyla level. Archaeal representatives in meaningful quantities (>0.5%) were found only in the samples of methane-containing water of the UC aquifer; their share in the communities of these waters comprised 7.9%–43.7% (Fig. 3).

In total, 688 phylotypes (amplicon sequence variants, ASVs) were revealed in all the water samples considering replicates. Of these, 642 phylotypes were identified only in a single well. At the genus level, the communities from three deep wells of the UC aquifer contained some common phylotypes, whereas the communities of the RA, as well as LC and UJ aquifers contained no common genera among each other, indicating near complete separation of microbial communities at different aquifers and recharge area (Table S9).

The RA microbial community was characterized by the highest biodiversity with uncultured taxa of the genus to class level representing over 70% of its composition. Dominant phylotypes belonged to the uncultured SRB2 family within the order *Thermoanaerobacterales* (17.2%) and the uncultured bacteria of the phylum-level group Sva0485 (11.5%). In addition, uncultured bacteria of the phyla *Nitrospirota* (8.1%) and *Spirochaetota* (5.0%) and of the family *Anaerolineaceae* (4.0%) had significant representation in this community (Fig. 3; Table S9).

The wells 49-E and 71 penetrating the deepest part of the UC aquifer, which bears Yessentuki no. 4 type mineral water, contained similar communities by their taxonomic composition (Fig. 2D and 3; Table S9), including the dominating (10%–45%) phylotypes of *Methanobacteriota* (former *Euryarchaeota*), uncultured *Aminicenantia, Coriobacteriia,* and actinomycetes of the RBG-16–55-12 group. A specific feature of the community profile retrieved from well 46, which bears Yessentuki no. 17 type mineral water, was the highest relative abundance of uncultured Archaea of the order *Hadarchaeales* (phylum *Hadarchaeota*), class *Thermoplasmata* (*Thermplasmatota*), and ANME-1a group (*Halobacteriota*). Together, these Archaea constituted more than 40% of 16S rRNA reads (Fig. 3; Table S9). In contrast, *Hadarchaeales* comprised 5.2% and 1.4% of the communities of wells 49-E and 71, respectively. *Methanothermobacter* phylotypes dominated among Archaea in both of these communities comprising 9.4% and 32.3% of their diversity, respectively. In addition, *Methanobacterium* phylotypes comprised 9.6% of the well 71 community but were absent in the community of the well 49-E, whereas *Bathyarchaeia* phylotypes comprised 2.6% in the well 49-E community but were among the minorities in the communities of wells 46 and 71 (Table S9).

In the LC aquifer, three phylotypes represented by uncultured bacteria of phylum *Nitrospirota*, order *Ignavibacteriales* (phylum *Bacteroidota*), and genus *Desulforudis* (phylum *Bacillota*) accounted for 78.6% of the community (well 9). A rather high representation of WPS-2 uncultured group should also be mentioned (Fig. 3; Table S9).

In the deepest UJ aquifer, a uniquely low diversity of prokaryotes was observed. A distinct dominance (more than 90% of 16S reads) of a phylotype belonging to the family *Thermoanaerobacteraceae* (Table S9) was observed in this community. Phylogenetic analysis revealed its close relatedness to *Aceticella autotrophica* (60). Such a community composition was stable over a 3-year sampling period (from September 2020 to April 2022) (Fig. 2D).

### Metabolic diversity and relative abundance of different physiological groups of prokaryotes in the microbial communities of the YMWB

A total of 213 metagenome-assembled genomes (MAGs) were assembled from all samples. Of these, 151 MAGs passed the quality check for their further comprehensive analysis (completeness >70% and contamination <5%), and 98 MAGs of them had completeness >90% and can be considered high-quality ones (Tables S2 and S3). Genome-resolved metagenomics generally supported the results of the 16S rRNA gene-based phylogenetic profiling and revealed an additional trend in the archaeal community succession from the predominance of *Hadarchaeota* to the prevalence of typical methanogens in the communities of UC aquifer with the increase of its depth from well 46 to well 71 (Table S3).

**Fig 4:**
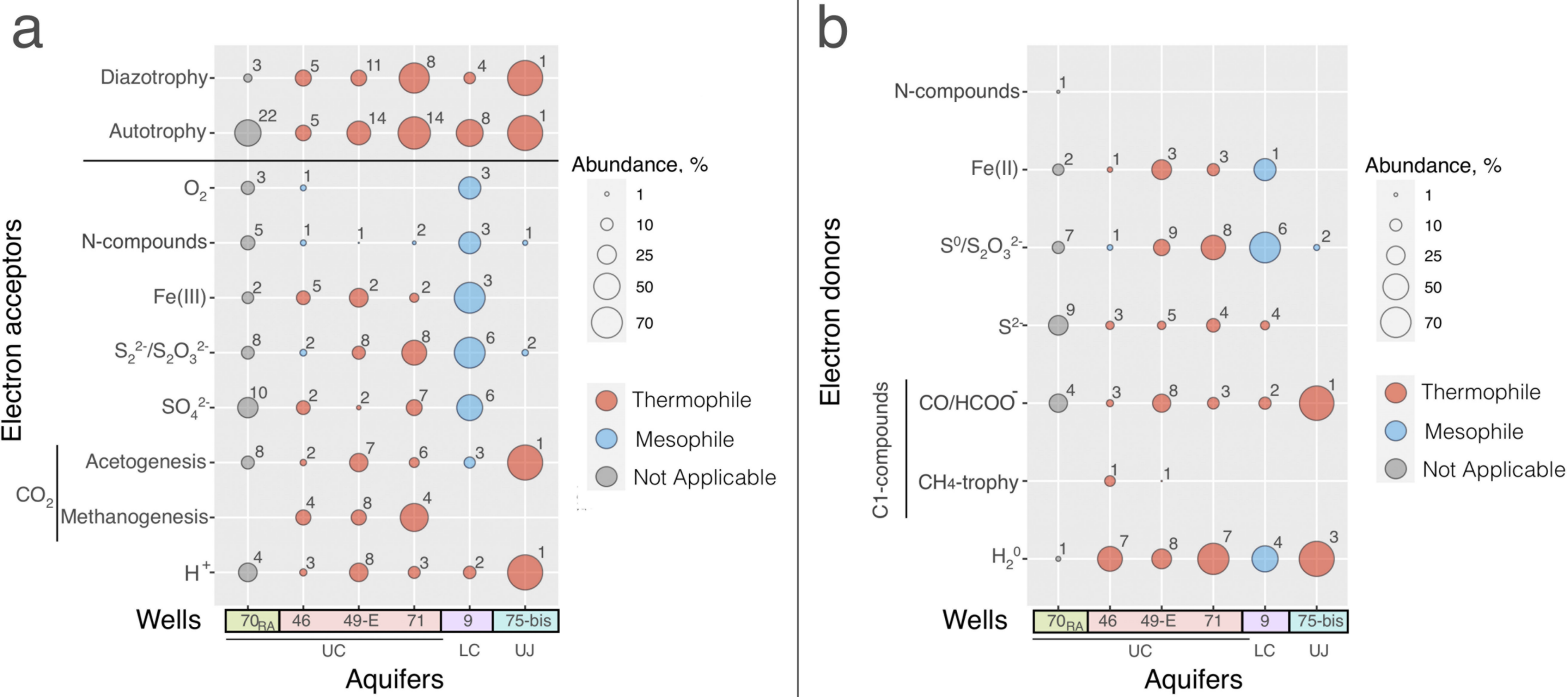
Metabolic diversity of the microbial communities of the YMWB aquifers as defined by metagenomic analysis. Representation of metabolic pathways for energy conservation and biomass formation in the MAGs assembled from all the sequenced metagenomes. (**a**) Pathways for carbon and nitrogen fixation and inorganic electron acceptors utilization. (**b**) Pathways for the utilization of inorganic electron donors. Algorithm of the screening for metabolic pathways is summarized in Table S5 and in Materials and Methods.

We screened the MAGs from all sampled wells for energy conservation pathways (utilization of various electron donors and acceptors), autotrophic carbon fixation, and diazotrophy. The majority of MAGs contained more than one of the screened metabolic pathways (e.g., the genes for iron and sulfur respiration, autotrophy, diazotrophy, etc., Tables S4 and S5). Metagenomic analysis revealed a broad representation of the pathways for CO_2_ and N_2_ fixation in all genotypes analyzed, with the proportion of diazotrophs increasing with aquifer depth (Fig. 4a). The maximum representation of autotrophs and diazotrophs was observed in the water of the deepest wells 71 and 75-bis (UC and UJ aquifer, respectively). The majority of the identified autotrophs possessed the Wood-Ljungdahl pathway for autotrophic carbon fixation (Fig. S5).

In terms of energy metabolism, anaerobically respiring organisms possessing the pathways for the utilization of sulfur, iron, and C_1_-compounds as electron acceptors or donors were highly represented in all sampled microbial communities (Fig. 4). In the UJ aquifer, hydrogenotrophic acetogens of a single genotype Yes75-02 (Table S4), related to *Thermoanaerobacteraceae*, clearly predominated (Table S3). Phylogenetic analysis revealed that it belongs to the dominant *Thermoanaerobacterium*-like phylotype (Table S9), representing the acetogen *A. autotrophica* (see above). In contrast, the organisms cycling iron or sulfur compounds predominated among anaerobes of the UC aquifer. Also, methanogens and putative anaerobic methanotrophs were detected only in this aquifer, and their share increased with its depth. The aerobic methane oxidation pathway and in particular the *amoABC* genes were not detected in any of the MAGs analyzed. The highest diversity of the respiratory pathways was revealed in the community metagenome of the upper part of the UC aquifer (well 46, Fig. 4). Notably, bacteria respiring N compounds constituted minor parts of all the analyzed populational genotypes except that of the well 9, whereas the organisms utilizing them as the energy sources were only identified in the RA community as its minor part (Fig. 4).

The share of putative H_2_-utilizing organisms detected in all the aquifers increased with depth, being maximal in the deepest part of UC and UJ aquifers (Fig. 4b). The absence or trace concentration of hydrogen in the gas phase of all the samples (Table 2) indicates that the communities act as efficient gas filters, significantly altering the composition of UC and UJ aquifer’s waters.

The highest proportion of putative aerobes was observed in the RA. Also, this physiological group was found in the water of LC aquifer. All the detected pathways for aerobic respiration were identified in the genotypes containing the pathways for anaerobic respiration as well (Table S4), thus representing the group of facultative anaerobes. The genotypes with several respiratory pathways, for example, sulfate and iron reduction, predominated in the warm oxidized waters of the RA and LC aquifer. In the last case, the organisms capable of sulfur, thiosulfate, iron, nitrate reduction, and aerobic respiration were mainly represented by only two genotypes, Yes09-04 and Yes09-11 (Table S4), of the family *Melioribacteraceae* and the order *Thermodesulfovibrionales*, which together comprised 65% of the population metagenomic reads (Table S3). In contrast, in the deepest part of the UC aquifer characterized by reduced conditions, redox transformations of sulfur and iron compounds were determined by different genotypes, that is, anaerobes of these ecosystems showed a narrow specificity for inorganic energy substrates and electron acceptors (Table S4).

### Most probable optimal growth temperatures of the organisms identified in the YMWB aquifers

Estimation of optimal growth temperatures from the amino acid content of the proteins encoded in each MAG revealed the prevalence of thermophilic organisms with optimal growth temperatures above 50°C in the UJ aquifer and in the submerged part of the UC aquifer, whereas mesophilic organisms predominated the community of the LC aquifer. These data correlate perfectly with the thermal anomaly of the YMWB, where the warm oxygenated aquifer is located in between two thermal reduced aquifers (Fig. 1). The mean estimated growth temperature optima for each of the microbial communities (Table S4) correlated with the aquifer water temperatures predicted by geothermometers (Table S6). The organisms able to use protons as the electron acceptors or reduced C_1_ compounds as the electron donors, as well as methanogens were represented exclusively by thermophiles (Fig. 4; Table S4).

### Primary enrichment cultures

**Fig 5:**
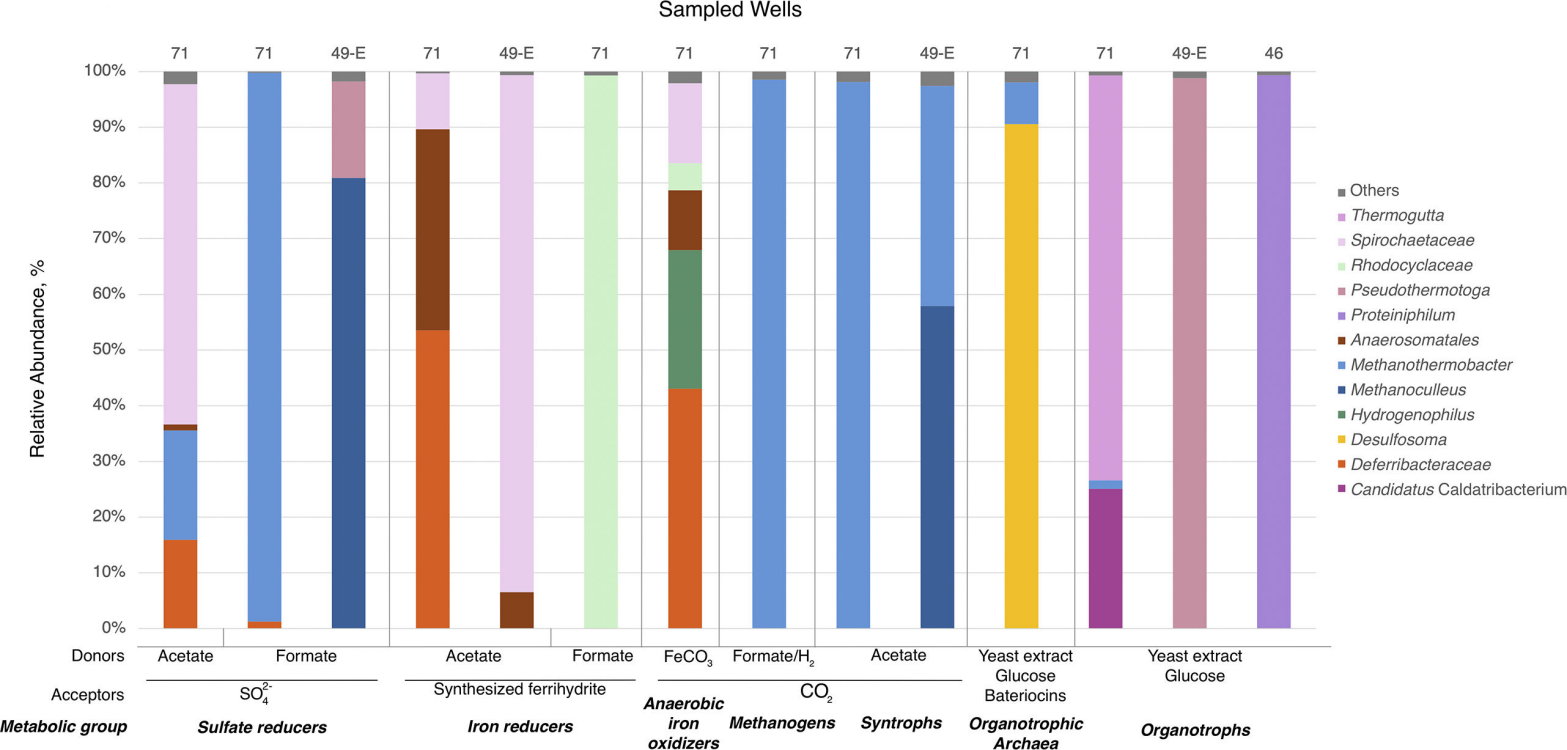
16S rRNA gene fragments profiling of the primary enrichments obtained from different water samples retrieved from the UC aquifer.

During culture-based microbiological studies, we obtained several enrichments that were further used for phylogenetic profiling and for the isolation of representatives of the major metabolic groups identified by metagenomic analysis (Fig. 5). Chemolithotrophic enrichment cultures from the UC aquifer grew much more rapidly than simultaneously incubated organotrophs and reached the stationary phase within few days versus ca. 3 weeks (Table S10; Fig. S6). Hydrogenotrophic methanogens of the genera *Methanothermobacter*, *Methanolinea*, and *Methanoregula* were isolated from the water of wells 71 and 49-E. Sulfate reducers were detected in the water of well 49-E but only in syntrophic associations with methanogens on a sulfate-free medium (Table S10). We isolated the strain of the genus *Thermodesulfovibrio* into the pure culture and observed its ability to reduce not only sulfate but also Fe(III) with formate. However, we failed to enrich hydrogenotrophic acetogens or any representatives of other metabolic groups from the UJ aquifer (Table S10). The most likely reason for the unsuccessful cultivation experiments is a significant difference in physico-chemical parameters (primarily pH and CO_2_ pressure) at the wellhead and in the deep subsurface reservoir of this aquifer.

Among the enriched metabolic groups, iron reducers from the water of the UC aquifer showed the fastest growth. Three novel representatives of this metabolic group were isolated from UC (well 71) and LC (well 9) aquifer water samples and published separately. A representative of the new family *Defferivibrionaceae* is an organotrophic iron-reducer able to oxidize a limited number of organic acids, but not formate (23). A representative of the previously uncultured order OPB41 (*Anaerosomatales* ord. nov., class *Coriobacteriia*, phylum *Actinomycetota*) predominating in the waters of the UC aquifer appeared to be a highly specialized obligately hydrogenotrophic Fe(III)-reducer (61). A representative of a new genus within the family *Melioribacteraceae* predominating in the water of the LC aquifer was a facultative anaerobe, respiring iron, sulfur, or arsenic compounds (62), thus being an example of a generalist species in the community.

## DISCUSSION

Continental subsurface environments increasingly attract the interest of researchers as intriguing testbeds related to the evolution of early life on Earth (63). The knowledge base on the inhabitants of these ecotopes is rapidly growing, but different environments and their microbial communities are characterized with different accuracy, periodicity, and repeatability, and corresponding papers often lack relevant geological and hydrogeochemical data (4, 10–12, 64–68). Microbial communities of subsurface drinking mineral water deposits are the least studied. Currently, researchers and subsurface users pay special attention to the sanitary control of natural drinking mineral water and to the microbiomes of bottled waters (69).

Our analysis of the geological and hydrogeochemical data summarized in the cross-section of YMWB highlights that this deposit bears a wide variety of water types within several spatially separated subsurface ecosystems in a relatively small area (Fig. 1 and Table 2; Fig. S3). Based on the correlation analysis of the physico-chemical parameters of the Yessentuki waters and the beta-diversity calculation for the microbial communities inhabiting them, we propose the presence of four distinct types of ecosystems in the studied area of the YMWB (Fig. 2). The analyzed microbial communities of these ecosystems have a low to negligible proportion of common taxa (Tables S3 and S9). Alpha-diversity calculations reveal the RA community to have the richest diversity and the UJ community to have the poorest one (Table S8). Highest standard deviation of richness for the RA site reflects the significant influence of its close connection to the ground surface on the community composition. Other analyzed communities are rather compact in terms of diversity, and in spite of small differences in their alpha-diversity characteristics (Table S8), they are clearly differentiated from each other depending on the physico-chemical conditions of their habitats. Such a differentiation of subsurface microbial communities reflected in their phylogenetic composition and predicted metabolic activity was previously reported for a CO_2_-driven geyser ecosystem. However, in that particular case, a significant proportion of common taxa found in different aquifers was revealed, due to the water mixing during the geyser eruptions (70).

The metabolic capacities of microorganisms inhabiting each of the four different types of YMWB subsurface ecosystems correlated with local characteristics of the physico-chemical setting. All analyzed microbial communities were dominated by anaerobes utilizing inorganic electron donors and acceptors (Fig. 4). Corresponding MAGs comprised 54%–97% of the total metagenomic reads in each of the communities (Tables S3 and S4). Hydrogen oxidizers were most widely represented among lithotrophs, which could be related to the steady upflow of juvenile hydrogen gas in deep aquifers (17). Alternatively, this energy source could be supplied via the interspecies hydrogen transfer in syntrophic processes of organic matter degradation. Energy conservation processes in all the communities were strongly dependent on the availability of inorganic compounds used for microbial growth, such as iron and sulfur minerals, CO_2_, etc. The inhabitants of the YMWB aquifers also showed a strong dependence on inorganic carbon and nitrogen sources, which increased with the depth of the aquifers, as evidenced by the increasing proportions of autotrophic and diazotrophic genotypes in the metagenomes of the UC and UJ aquifer communities (Fig. 4a). This was further supported by a prevalence of chemolithotrophic prokaryotes over the organotrophs in primary enrichments obtained from UC aquifer (Fig. 5; Table S10). Rapid enrichment of lithotrophic hydrogenotrophic iron(III) reducers and methanogens from the water of this aquifer correlated well with the results of metagenomic analysis which revealed high representation of these metabolic features in the UC community metageomes (Fig. 4; Table S4).

The majority of autotrophs identified in all the metagenomes possessed the Wood-Ljungdahl pathway (Fig. S5), the most ancient and the only energy-releasing route of biological CO_2_ fixation (71). Among the genotypes encoding the Wood-Ljungdahl pathway, those of methanogenic archaea occurred only in the UC aquifer (Fig. 3 and 4), where the water contained CO_2_ but had a lower gaseous factor than in the UJ aquifer (Table 2). The metabolic activity of methanogens was confirmed by the biogenic origin of isotopically light methane (Table S7) in the free gas phase of the exposed waters. It could be one of the factors leading to the decrease in CO_2_ content of the UC aquifer compared with that of the UJ (Table 2). The reverse situation was observed in the deepest UJ aquifer where hydrogenotrophic acetogens *A. autotrophica*, genotype Yes75-02, outcompeted all the other hydrogen-utilizing groups of prokaryotes (Fig. 4; Tables S3 and S4). Even the relatively high sulfate conсentration in the water of this aquifer did not lead to the proliferation of sulfate reducers. Such an unusual selection for the organisms possessing the least thermodynamically favorable pathway for energy conservation could be explained by a water supersaturation with CO_2_ and lowered pH (calculated value 5.5, Table 2). The pH and temperature parameters calculated for this aquifer (Table 2) were consistent with the growth optima of the *A. autotrophica* type strain (60).

The phylogenetic and metabolic diversity of the YMWB microbial communities correlated with the proximity of their host aquifers to the ground surface and with the gas-hydrochemical mode of the groundwater (Table 2). The influence of aquifer hydrodynamics on microbial diversity was evidenced by the difference in the metabolic capacities of dominant physiological groups of the RA and LC aquifer versus those of deeper, more reduced, and hydrodynamically stable UC and UJ aquifers. Apparently, a low water exchange rate (Fig. 1; Table 2) maintained the physico-chemical stability of deeper ecosystems and stimulated the selection of highly specialized metabolic groups instead of generalistic ones.

The small thickness of the sedimentary cover, the proximity of magma sources to the aquifers, the presence of tectonic faults and intrusions formed during Alpine folding, and, finally, a steady influx of gas and water fluids that actively interact with the sedimentary rocks (17, 18) caused considerable similarities between physico-chemical settings of YMWB aquifers and some environments of the ancient Earth (13, 14). The structure of the four proposed subsurface ecosystems of YMWB was determined by a combination of factors that were secondarily superimposed on the Upper Jurassic and Cretaceous sedimentary rocks during the Alpine folding period (72). This millennia-long impact was the driving force for the formation of several distinct microbial communities whose metabolic properties might resemble those thought to dominate the Earth’s prokaryotic biosphere during three critical periods of its evolutionary history.

The ecosystem of the deepest UJ aquifer with the predominance of thermophilic, hydrogenotrophic, homoacetogenic, autotrophic, and diazotrophic bacterium *A. autotrophica* resembles the proposed earliest ecotopes of the Earth formed under high partial pressure of carbon dioxide, high magmatic activity, and reduced environment that favored the evolvement of hydrogenotrophic acetogenesis (58, 73–75). Evolutionary reconstructions predict the last universal common ancestor (LUCA) and its first descendants, which lived approximately 3.4 billion years ago, to be capable of autotrophic carbon fixation via the Wood-Ljungdahl pathway, that is, via acetogenesis coupled to energy conservation, and to be thermophilic anaerobes (76, 77). It was suggested that such microorganisms might have played a key role as the primary producers of organic matter in the early stages of the Earth’s biosphere history (78), but to date, no modern econiches were found, in which this process dominated. Our studies close this knowledge gap by describing a habitat, under physico-chemical parameters in which hydrogenotrophic acetogenic bacteria could act as a community-fueling group.

The conditions in the UC aquifer correspond to those of the geological period of banded iron formations (BIF) accumulation in anoxic CO_2_-rich environments 3.2–2.4 billion years ago, when biogeochemical cycles of carbon and iron prevailed over the sulfur cycle, and the processes of acetogenesis, methanogenesis, iron reduction, and anaerobic iron oxidation were much more active than the biogenic sulfidogenesis (79–84). The UC aquifer has high but unsaturated CO_2_ content in the free gas phase, which shapes the metabolic diversity of its microbial population toward the predominance of autotrophic methanogenic *Archaea*, which are considered to be the ancestors of all currently known organisms of this domain (85). Our estimation of optimal growth temperatures from metagenomic data indicated that all identified methanogens are thermophiles, as generally proposed for ancestral archaeal forms (Fig. S5). In addition to methanogens, a comprehensive representation of iron- and sulfur-cycling prokaryotes was revealed in the UC aquifer (Fig. 4). The relative abundance of these metabolic groups resembles the proposed structure of microbial communities whose activity could contribute to the deposition of BIF. Our culture-based studies well confirmed the active development of litho- and organotrophic iron-reducing bacteria from UC samples (Fig. 5; Table S10).

Finally, the unstable conditions in the studied section of the LC aquifer with the oxygen present in the water are reminiscent of the period of oxygen appearance in the Earth’s atmosphere (great oxidation event, GOE), which occurred approximately 2.4 billion years ago (86, 87). At the beginning of this period, generalist species of metabolically versatile prokaryotes could outcompete highly specialized obligate anaerobes. Similarly, the microbial community of oxygenated LC aquifer of YMWB is predominated by facultative anaerobes with flexible respiratory metabolism (Fig. 4; Table S4). For example, representatives of the family *Melioribacteraceae*, which constitute a significant part (23%) of the LC microbial community, are capable of oxygen, Fe(III), and thiosulfate respiration (88, 89). The predominance of “multifunctional” microbial groups in the LC aquifer of YMWB is in agreement with its hydrodynamic and hydrochemical parameters, indicating active water exchange in an area located far from the recharge area (Fig. 1).

**Fig 6:**
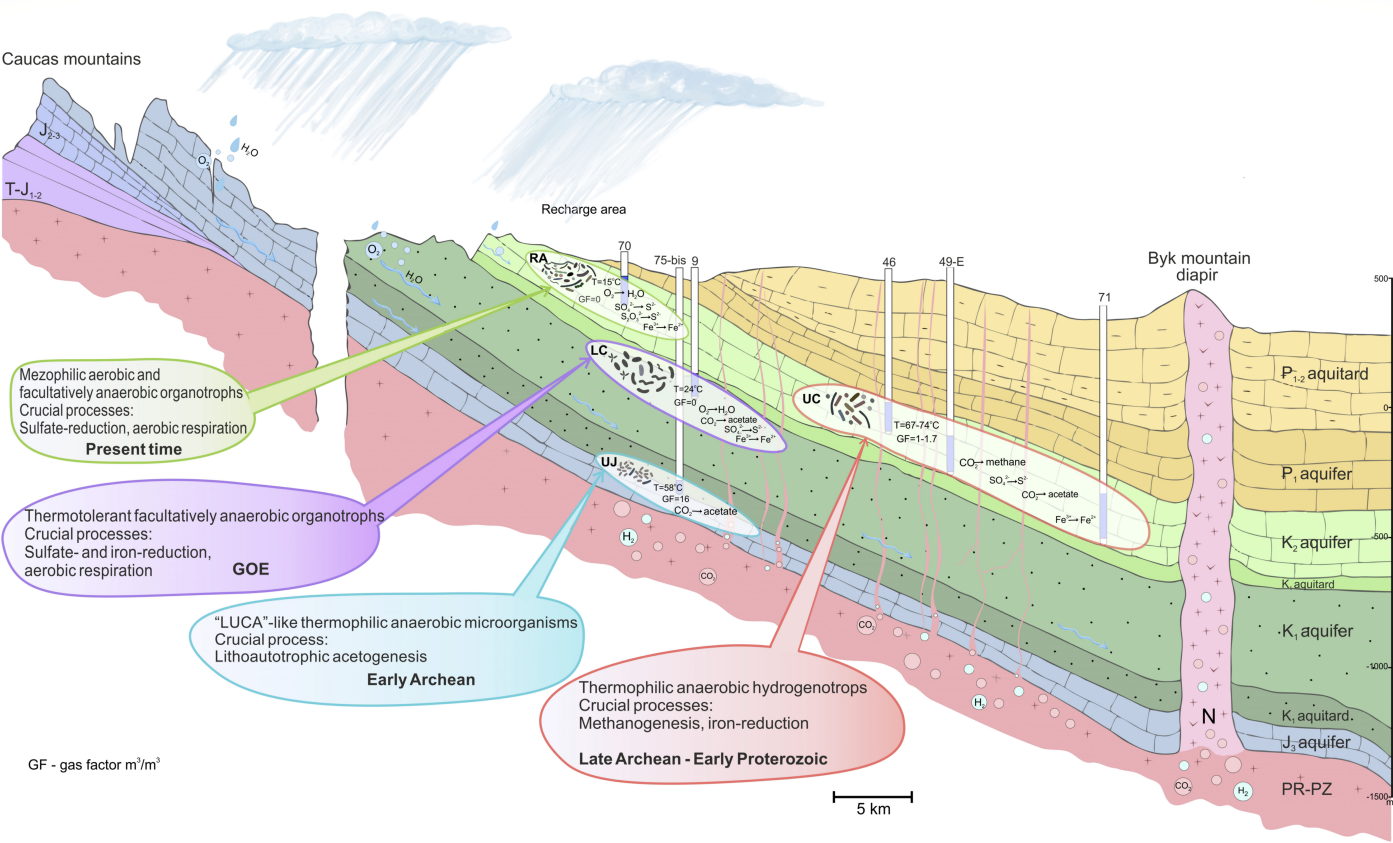
Graphical summary of the proposed ecosystems of the YMWB aquifers penetrated by the studied wells and their relation to the crucial junctures of the Earth’s biosphere history.

Totally, we have revealed several modern ecosystems in which similar physicochemical conditions compared with three crucial junctions of Earth’s history favored *de novo* formation of the microbial communities presumed to dominate the early Earth (Fig. 6). Two of them, the BIF accumulation and GOE period, have been already shown to be modeled by modern ecosystems. For example, some hydrothermal ecosystems on Milos (90) or Shikinejima (91, 92) islands are considered to be the modern analogs of BIF. Physico-chemical conditions characteristic of the GOE are widely reproduced on modern Earth in sedimentary environments that are equally influenced by biogeochemical cycles of iron and sulfur. Here, we describe the first example of an ecosystem in which a LUCA-like acetogenesis-based microorganism could have evolved. The discovery of such an ecosystem in a geologically modern formation encourages new exploration of the deep subsurface to uncover the evolutionary traits in microbial populations that may have played a critical role in the formation of the Earth’s biosphere.

## Data Availability

All sequencing data were deposited in the NCBI SRA database under BioProject numbers PRJNA760784 and PRJNA1083965. All MAG assemblies were deposited in GenBank under BioProject numbers PRJNA967334 and PRJNA958107.

## SUPPLEMENTAL MATERIAL

Supplemental material mbio.02831-24-s0001.docx Supplemental figures and tables.

Table S3 mbio.02831-24-s0002.xlsx General characteristics of the MAGs.

Table S4 mbio.02831-24-s0003.xlsx Representation of metabolic pathways.

Table S9 mbio.02831-24-s0004.xlsx Phylogenetic diversity of YMWB microbial communities.

Fig S5 mbio.02831-24-s0005.xlsx Heat map indicating the capabilities for inorganic carbon fixation encoded in the MAGs assembled from YMWB community metagenomes.

ASM does not own the copyrights to Supplemental Material that may be linked to, or accessed through, an article. The authors have granted ASM a non-exclusive, world-wide license to publish the Supplemental Material files. Please contact the corresponding author directly for reuse.
