# Supplementary material for: Analogs of Precambrian microbial communities formed de novo in Caucasian mineral water aquifers
Source: mBio. 2024 Dec 11;16(1):e02831-24. doi: 10.1128/mbio.02831-24 (PMC11708057; doi:10.1128/mbio.02831-24)
Supplement: Supplemental material — Supplemental figures and tables. [file mbio.02831-24-s0001.docx]

**Analogs of Precambrian Microbial Communities Formed *de novo* in Caucasian Mineral Water Aquifers**

Daria G. Zavarzina^a^, Alexey A. Maslov^b^, Alexander Y. Merkel^a^, Nataliya A. Kharitonova^b^, Alexandra A. Klyukina^a^, Ekaterina I. Baranovskaya^b^, Elena A. Baydariko^b^, Evgeniy G. Potapov^c^, Kseniya S. Zayulina^a^, Andrey Y. Bychkov^b^, Nikolay A. Chernyh^a^, Elizaveta A. Bonch-Osmolovskaya^a,d^, Sergey N. Gavrilov^a^

**SUPPORTING INFORMATION**

***Figure S1.*** Design of water sampling spot at a wellhead of a YMWB production well.


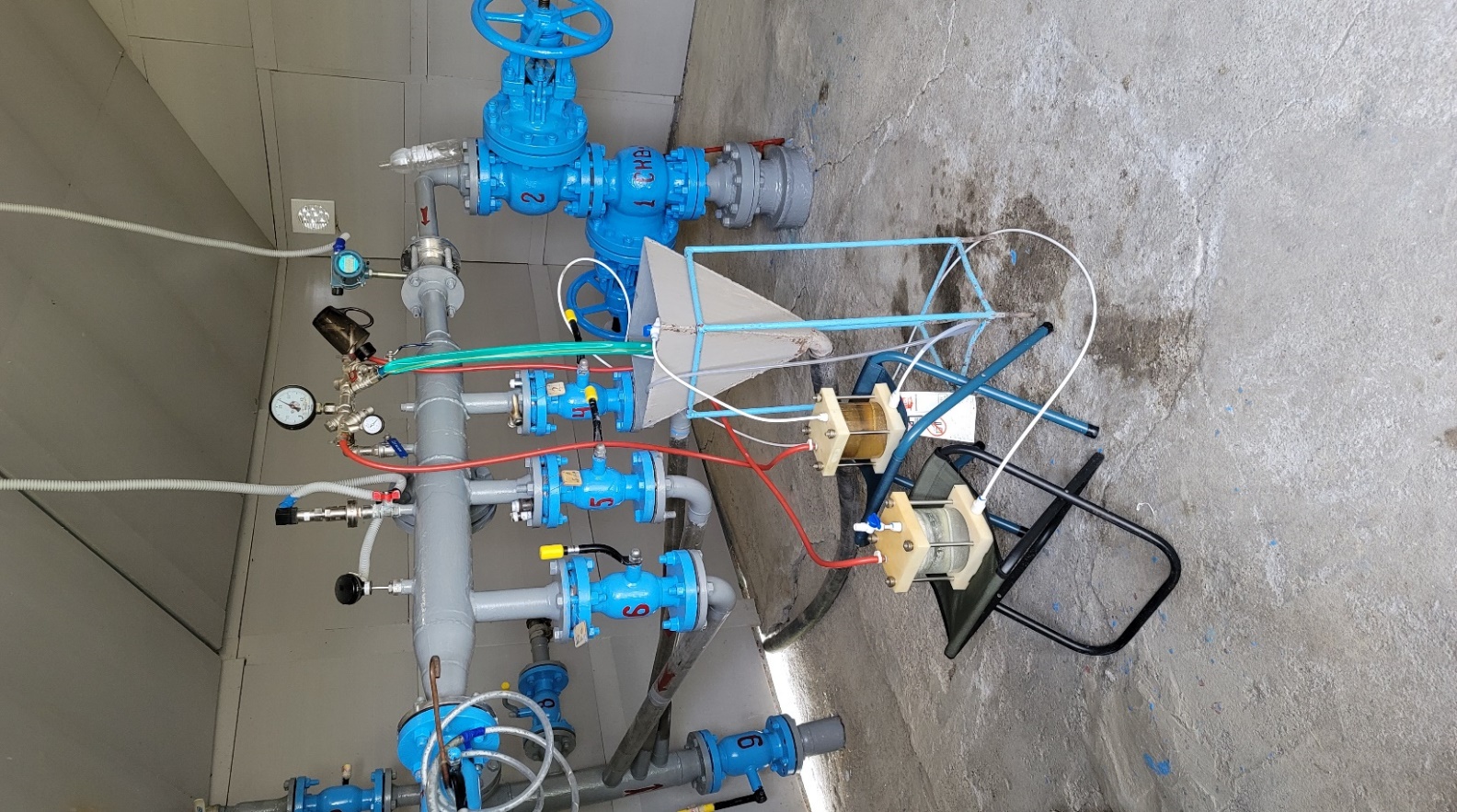

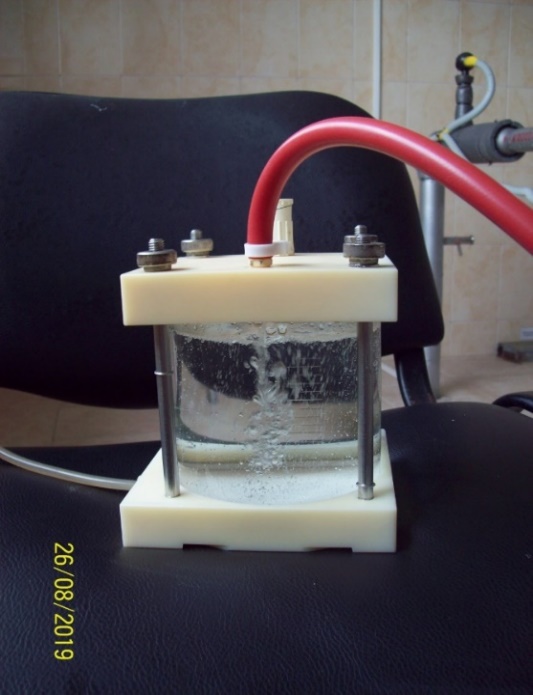

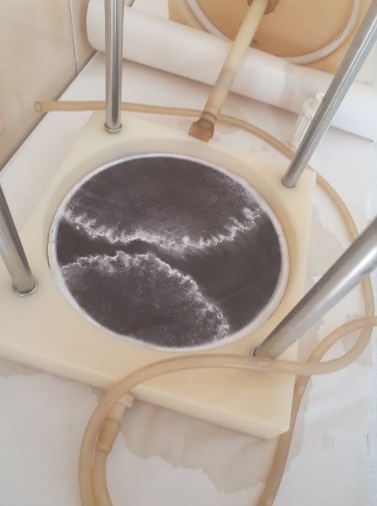


Ultrafiltration units

FM02-1000

track membrane filter after 100 L water filtration

Sterile pressure control unit

Wellhead

***Figure S2.*** Location of the wells studied along the hydrogeological cross-section line of YMWB (see Fig. 1 for the cross-section).

**
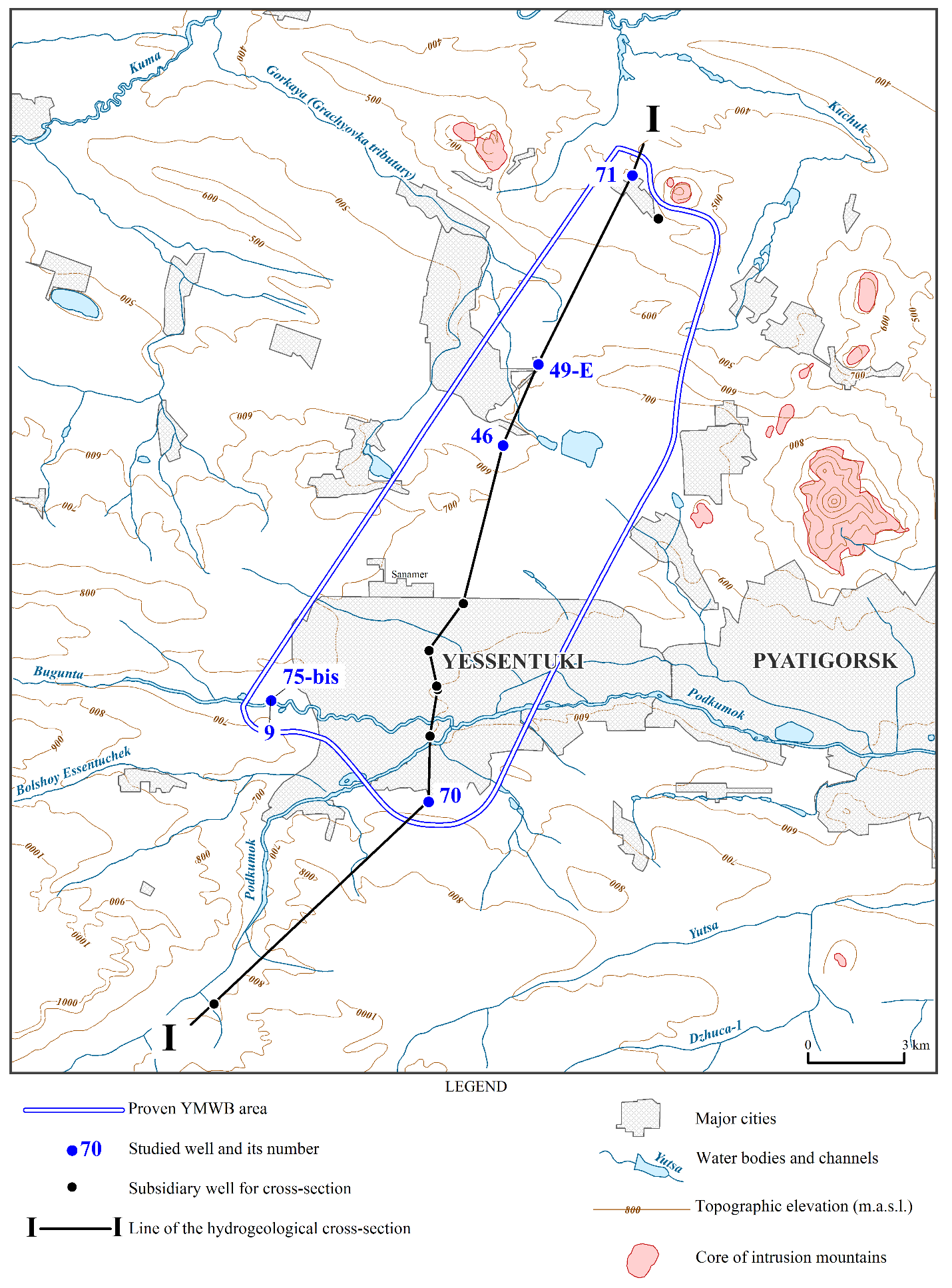
**

***Figure S3.*** Ternary plots for cations (Ca^2+^-Mg^2+^-Na^+^) and anions (HCO_3_^-^-SO_4_^2-^-Cl^-^) content of groundwater sampled from the wells penetrating UC (46, 49-E, 71), LC (9), and UJ (75-bis) aquifers. Well 70 penetrates the recharge area (RA) of the UC aquifer.

**
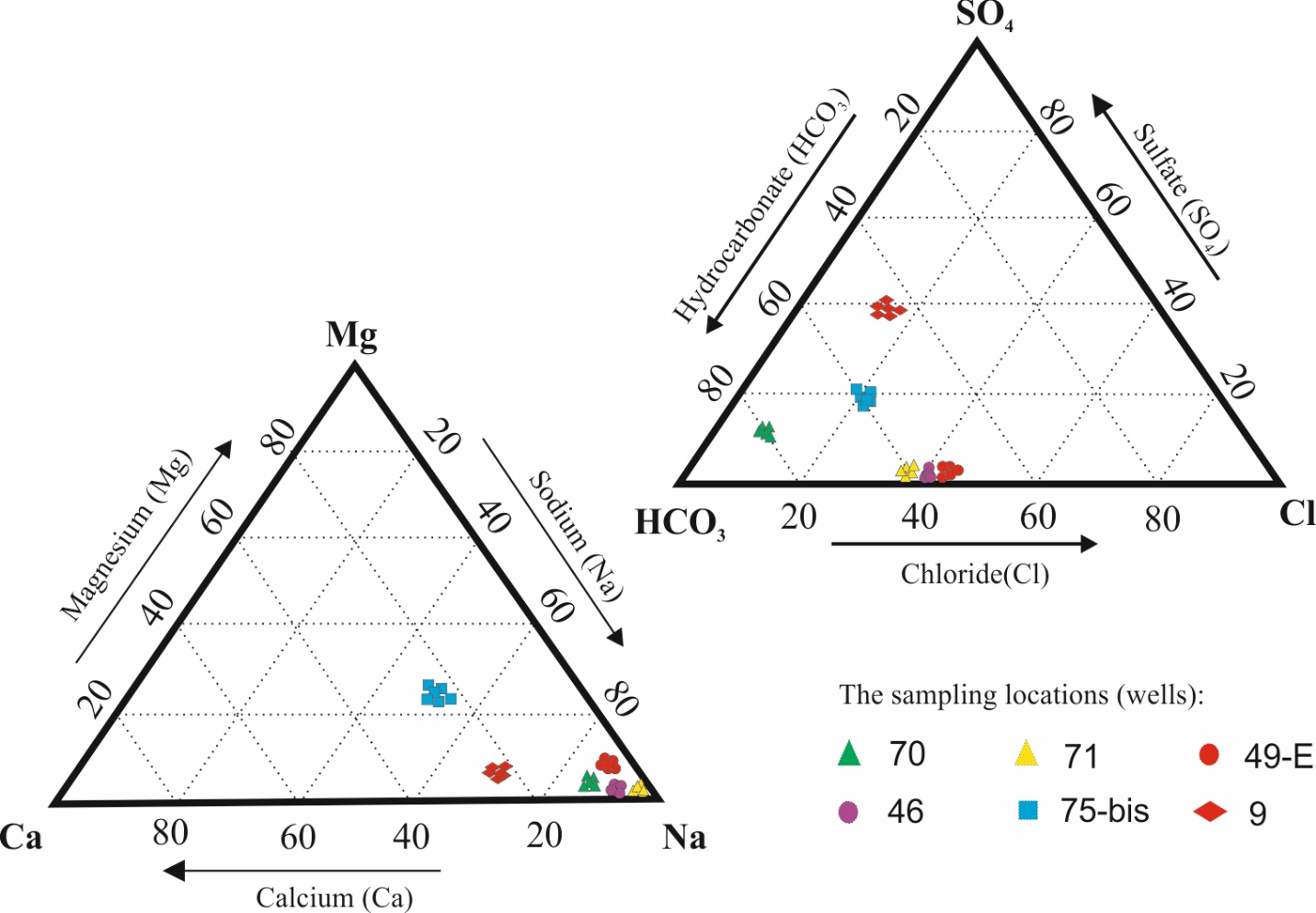
**

***Figure S4.*** Binary diagram of δ^18^О_SMOW_ and δD_SMOW_ isotopic composition of natural mineral waters of the YMWB.


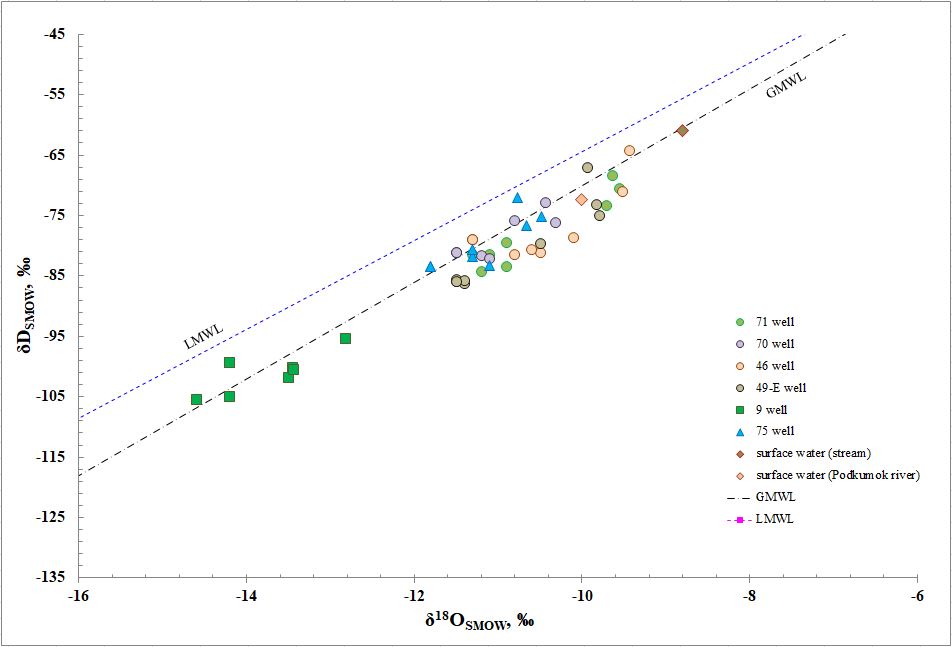


The stable isotope ratios in the investigated samples show significant variations of δ^18^O and δD. The δ^18^O and δD values of the groundwater samples range from -14.6‰ to -9.4‰ and from -105.4 to -64.2‰, respectively. Water from the K_1_*a-al_1_* (LC) aquifer has more negative values (about 3‰ for δ^18^O and 15‰ for δD) than the other groundwaters. The Global Meteoric Water Line (GMWL, Craig, 1961) and the Local Meteoric Water Line (LMWL) are shown for reference. We have used the data from the nearest Global Network for Isotopes in Precipitation (GNIP) station at Batumi, Georgia, to establish the Local Meteoric Water Line (IAEA/WMO, 2006). Global Network for Isotopes in Precipitation. The GNIP Database. <http://www.iaea.org/water>.

All the data obtained lie between the GMWL and the LMWL, indicating that all the groundwaters are of meteoric origin. The pCO_2_ in the groundwater does not seem to influence the δ^18^O and δD values; therefore, we can use these data to evaluate the recharge altitudes of the groundwater. The groundwater isotopic values are similar to those of the river water near the wells. The groundwater samples from the LC aquifer show more significant depletion in δ^18^O and δD values (difference up to 3‰ and 20‰ respectively) compared to waters from the K_2_*s-m* (UC) and J_3_*tt*-K_1_*v* (UJ) aquifers.

Based on the relationship between δ^18^O values and precipitation altitude (depletion 2‰ per 100 m), the recharge altitude of meteoric water can be estimated. The average recharge altitudes of the groundwaters studied were calculated as follows: 650-680 m asl for the UJ aquifer; 1000-1100 m for the LC aquifer; and 600-630 m for the UC aquifer. Thus, the groundwater from the UC aquifer was recharged at the same altitude as the UJ aquifer, but the recharge area of the LC aquifer was almost 400 m higher (or more part of the winter recharge).

***Figure S5.*** Heat map indicating the capabilities for inorganic carbon fixation encoded in the MAGs assembled from YMWB community metagenomes. Presented as a separate MS Excel file.

***Figure S6.*** Micrographs of primary enrichments at their stationary growth phases, obtained with fluorescent acridine orange dye (a-c) or by phase-contrast microscopy (d-f): (a) organotrophic and (b) lithotrophic iron reducers from well 71; (c) lithotrophic methanogens of the genus *Methanothermobacter* from well 49-E; (d) syntrophic acetate oxidizers from well 71; (e) organotrophs of the genus *Pseudothermotoga* from well 49-E; (f) organotrophs from well 71 belonging to the genera *Thermogutta* and ‘*Candidatus* Caldatribacterium’. Bar=10 µm.


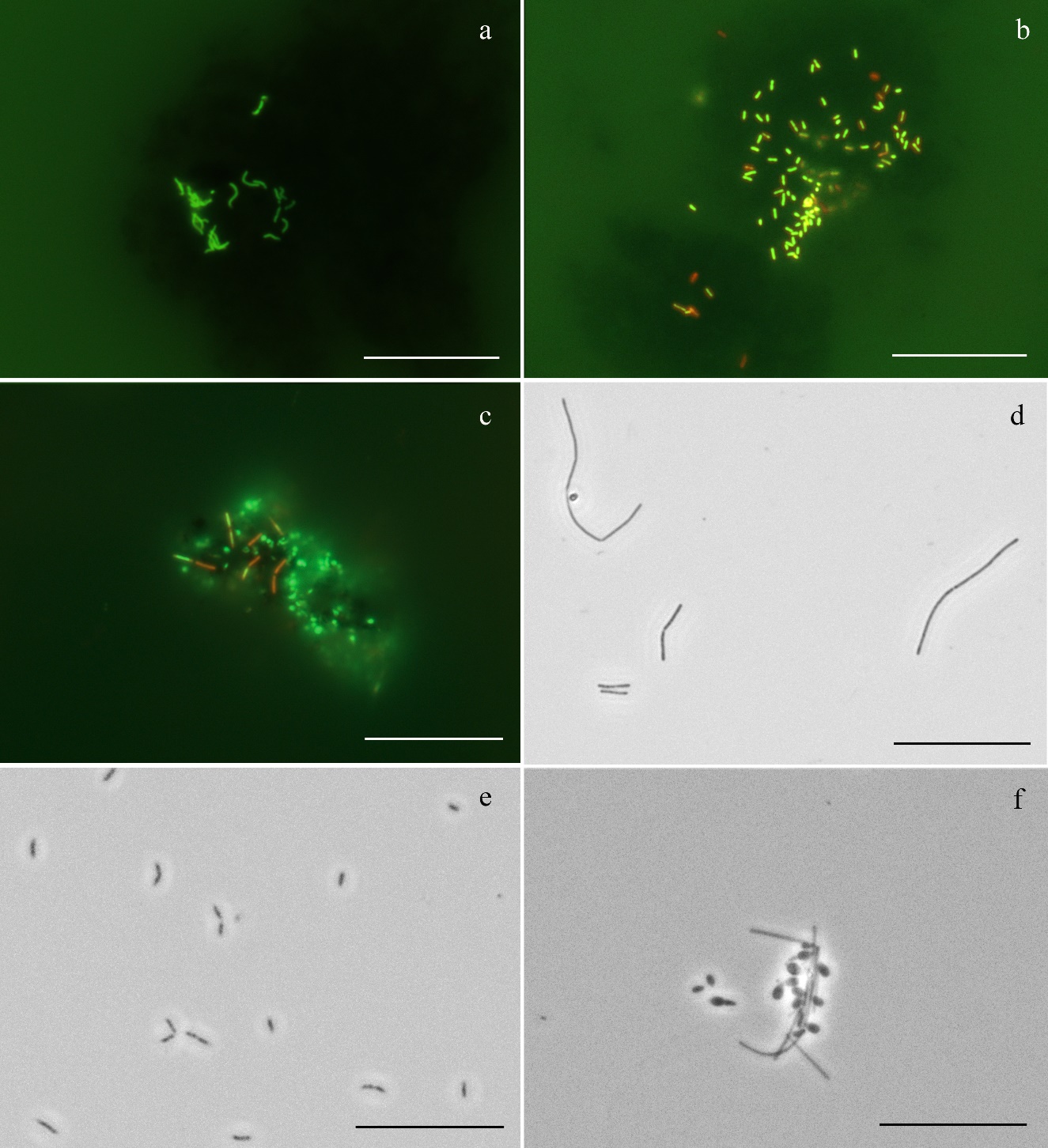


***Table S1.*** Characteristics of the sampled wells. The left column is colored according to beta-diversity groups from Figure 2.

| Well No/ Abbreviation | Coordinate | Aquifer/ Abbreviation | Sediments | Depth, m | Open borehole at, m | Days of operation before sampling | Borehole volumes extracted before sampling | The borehole volume**, m^3^ | Time of one borehole volume extraction, hours | Sampling dates,  DD.MM.YY |
| --- | --- | --- | --- | --- | --- | --- | --- | --- | --- | --- |
| 70/ *RA (recharge area)* | E 42°51'50" N 44°0'50" | Upper-Cretaceous K_2_*s-m*/  UC | limestones, marls, and bundles of sandstone in the roof and base | 212.0 | 81.0-212.0 | 218 | 10166 | 1.46 | 0.51 | 07.09.2020 30.11.2020 (twice a day) |
| 46/UC | E 42°53'35" N 44°6'50" | Upper-Cretaceous K_2_*s-m*/  UC |  | 685.8 | 552.0-685.8 | 229 | 4420 | 3.32 | 1.25 | 23.07.2019* 08.09.2020 29.11.2020 (twice a day)  16.04.2022 |
| 49-E/UC | E 42°54'25" N 44°6'50" | Upper-Cretaceous K_2_*s-m*/  UC |  | 865.0 | 580.0-865.0 | 228 | 3091 | 5.83 | 1.77 | 23.07.2019*, 08.09.2020 29.11.2020 (twice a day) 16.04.2022 |
| 71/UC | E 42°56'30" N 44°11'20" | Upper-Cretaceous K_2_*s-m*/  UC |  | 998.9 | 676.0-998.9 | 293 | 1804 | 7.80 | 3.90 | 07.09.2020 30.11.2020 (twice a day) |
| 9/LC | E 42°48'10" N 44°2'30" | Lower-Cretaceous *K_1_*a-al_1_/  LC | sandstones and siltstones with interlayers of clay bundles | 600.0 | 485.0-556.0 | 204 | 360 | 13.38 | 13.38 | 09.09.2020 28.11.2020 (twice a day) |
| 75-bis/UJ | E 42°48'10" N 44°2'30" | Upper Jurassic  *J_3_*tt*-K_1_*v/  UJ | K_1_ limestones and J_3_ red terrigenous sediments, up to 200 m thick. | 974.0 | 943.0-974.0 | 206 | 223 | 14.81 | 22.2 | 09.09.2020 28.11.2020 (twice a day)  17.04.2022 |

*(Gavrilov et al., 2022)

** The borehole volume of each well is calculated from its geological and technical profile: for each interval of different diameter its volume is obtained as the product of area by length, then all interval volumes are summed for each well.

***Table S2.*** General characteristics of the results of metagenomic sequencing and contig assembly. Wells are colored according to beta-diversity groups from Figure 2.

| Aquifers | Wells | Total # reads (2x100) received | # contigs | Total length, bp | Largest contig, bp | N50 | L50 |
| --- | --- | --- | --- | --- | --- | --- | --- |
| **UC** (*RA*) | **70** | 254057484 | 47693 | 185127208 | 532221 | 5562 | 6780 |
| **UC** | **46** | 29046046 | 27203 | 76684406 | 510237 | 7210 | 1875 |
|  | **49-E** | 46624961 | 22419 | 116860009 | 1817926 | 11610 | 1449 |
|  | **71** | 81583831 | 13896 | 89212251 | 1170803 | 14887 | 886 |
| **LC** | **9** | 129330736 | 13794 | 80772497 | 1241385 | 18864 | 567 |
| **UJ** | **75-bis** | 21610388 | 5928 | 29917449 | 144982 | 8123 | 711 |

***Table S3.*** General characteristics of the MAGs from water samples of YMWB which passed the quality check. Presented as a separate MS Excel file.

***Table S4.*** Representation of metabolic pathways for energy generation and biomass formation in the MAGs assembled from all the sequenced metagenomes and passed the quality check. Presented as a separate MS Excel file.

***Table S5.*** Algorithm of the screening for metabolic pathways of biomass formation and energy conservation in the MAGs assembled from all the sequenced metagenomes. The pathways are given in accordance with the Table S4.

For each of the metabolic features several alternative KEGG modules and/or gene sets were screened for in the MAGs which passed the quality check. Key proteins and genes of the modules and custom gene sets are listed in the table using Boolean operators: AND means that both of the genes/proteins should be present in a MAG to assign the target feature to the organism, OR means that any of the mentioned entries was considered to determine the target metabolic feature. Groups of several alternative key proteins determining a metabolic feature are given in brackets and marked yellow or green for clarity.

| **Target metabolic features** | **Target KEGG modules and gene sets** | **Completeness of a module or gene set, %** | **Key proteins, necessary and sufficient to assign a pathway** | **Cutoffs for key proteins, identity/query coverage, ≥%** | **Refs. for the IDs of query gene sequences** | **Source database of query sequences** |
| --- | --- | --- | --- | --- | --- | --- |
| Autotrophic carbon fixation**^1^** | Calvin–Benson–Bassham cycle (M00165), Wood–Ljungdahl pathway (Reductive Acetyl CoA, M00377), reductive TCA (Arnon-Buchanan) cycle (M00173), 3-Hydroxypropionate bi-cycle (M00376), Dicarboxylate-4-Hydroxybutyrate cycle (M00374), 3-hydroxypropionate-4-hydroxybutyrate cycle (M00375), reductive glycine pathway**^1^**, reverse oxidative TCA (roTCA) cycle**^1^** | 80-100 | catalytic subunits of the enzyme complexes included in the modules | 50 / 80 | KEGG database  Steffens et al., 2022 (for roTCA cycle),  Sánchez-Andrea et al., 2020 (for the reductive glycine pathway) | KEGG database  UniProt Knowledgebase |
| Diazotrophy | nitrogen fixation (M00175) | 80 | NifDKH | 50 / 80 | Simon & Klotz, 2013 | UniProt Knowledgebase |
| Dissimilatory reduction of nitrogen compounds | denitrification (M00529),  nitrate reduction (M00530) | 80 | NarGHI **OR** NapAB **OR** NirK **OR** NirS **OR** NorBC **OR** NosZ | 50 / 80 | Simon & Klotz, 2013 | UniProt Knowledgebase |
| Oxidation of nitrogen compounds | nitrification – ammonia to nitrite (M00528) or to nitrate, commamox (M00804), anammox (M00973) | 80 | [AmoCAB **AND** Hao]  **OR** [Hzs **AND** Hzo] | 50 / 80 | Simon & Klotz, 2013 | UniProt Knowledgebase |
| Methanogenesis | acetoclastic (M00357), or hydrogenotrophic (M00567), or methylotrophic (M00563, M00356) methanogenesis | 80 | catalytic subunits of all the enzyme complexes included in the modules | 60 / 80 | KEGG database | KEGG database |
| Methanotrophy | aerobic methane oxidation (M00174)^*^,  or ANME metabolic patterns | 80 | AmoABC^*^ **AND** [Mdh1/ MxaF (EC:1.1.2.7)]  **OR** [Mcr **AND** Fqo/Fpo **AND** Hdr] | 60 / 80  60 / 80 | KEGG database  Timmers et al., 2017 (for ANME genes) | KEGG database  Custom gene database **^2^** |
| Acetogenesis | Wood-Ljungdahl pathway (M00377),  CO to acetate pathway **^3^**:  M00377 AND Acs (EC 2.3.1.169) AND energy converting hydrogenase (ECH, EC 1.12.7.2), or Rnf complex (COG4658), or Hyd4b hydrogenase AND [Ni,Fe]-CO-dehydrogenase (CODH) AND bifurcating hydrogenase | 80-100  100 | catalytic subunits of all the enzyme complexes in M00377,  80-100% complete M00377 module **AND** Acs (acetyl-CoA synthetase) **AND** [ECH **OR** RnfD **OR** Hyd4b] **AND** CODH (CooS subunit) **AND** bifurcating hydrogenase | 60 / 80  30**^4^** /80 | KEGG database  Schoelmerich & Müller, 2020 | KEGG database  UniProt Knowledgebase |
| CO/formate-trophy | Wood-Ljungdahl pathway (M00377) AND CODH/Hyd4b AND ECH, EC 1.12.7.2 | 100 | 80-100% complete M00377 module **AND** **AND** [ECH **OR** Hyd4b] **AND** CODH (CooS subunit) | 30**^4^** / 80 | Kim et al., 2010 (for formate-trophy); Schoelmerich & Müller, 2020, and references therein | KEGG database  UniProt Knowledgebase |
| H^+^ reduction | ECH or Hyd4b AND M00377 AND CODH | 80-100 | [ECH **OR** Hyd4b complexes] **AND** 80-100% complete M00377 module **AND** CODH | 30**^4^** /80 | KEGG database (for M00377), Schoelmerich & Müller, 2020 | KEGG database  UniProt Knowledgebase |
| H_2_ oxidation | Hyb (EC 1.12.99.6),  Hox (EC 1.12.1.2), Fe-only hydrogenase (EC 1.12.7.2), Ech (UniProt entry A0A0G3CGQ5), Mbh (UniProt entry A2SS19), Mvh, Vht, Frh, Hmd (EC 1.12.99.-, 1.12.98.3, 1.12.98.1, and 1.12.98.2, respectively) | >50 of any of the set | HybOC **OR** HoxFUY **OR** HydA **OR** EchABE **OR** MbhHJK **OR** MvhAD **OR** VhtAC **OR** FrhAB **OR** Hmd | 60 / 80 | Greening et al., 2016 and references therein | UniProt Knowledgebase |
| Sulfate reduction | M00569 | 80-100 | Subunits of the complexes:  Sat (EC 2.7.7.4) **AND** AprAB (EC 1.8.99.2) **AND** DsrAB (EC 1.8.99.5) **AND** DsrC (EC 1.8.1.22) **AND** QmoABC | 60 / 80 | Chernyh et al., 2020 (including archaeal Dsr) | UniProt Knowledgebase |
| Sulfur & thiosulfate reduction and disproportionation | Psr/Phs complexes (KEGG orthologs K08352 & K08354), or  SseA AND GlpE (KEGG orthologs K01011 & K02439) | 100  100 | PsrAB **OR**  PhsAB  SseA **AND** GlpE | 60 / 80  60 / 80 | Sabuda et al., 2020 | UniProt Knowledgebase |
| Sulfide, sulfur, and thiosulfate oxidation | SOX complex (KEGG orthologs K17222, 17223, 17224, 17225, 17226, 17227), thiosulfate oxidation to tetrathionate (K19713, 16936, 16937), sulfide-quinone oxidase (K17218), sulfide dehydrogenase (K17220, 17230) | 80-100 | SoxBC **OR** DoxAD **OR** TdsA **OR** FccAB **OR** Sqr | 60 / 80 | Sabuda et al., 2020 | UniProt Knowledgebase |
| Fe(III) reduction | Multiheme cytochromes (PS51008 PROSITE Multiheme cytochrome *c* family proteins) and type IV pilus assembly proteins (PF07963) and alike archaeal flagellins with reported involvement in extracellular electron transfer in prokaryotes – totally, 63 entries of a custom database | ≥5  (at least, 1 membrane-associated multiheme cytochrome AND 1 quinol-oxidizing AND 1 auxiliary electron shuttling cytochrome) | [MtrC/MtrF **OR** OcwA/OmhA **OR** OmcC/OmcS/OmcZ **OR** any of archaeal putative EET-related multihemes with UniProt entries: AIY90869.1, ADC65421.1, PXF61242.1, ALL00834.1] **AND** one of CymA/Cbc or Pcc complexes | 20 **^5^** / 70 | Gavrilov et al., 2017; 2021; Toshchakov et al., 2018 and references therein (for multihemes of meso- & thermophilic Fe(III) respiring bacteria), Mardanov et al., 2015; Smith et al., 2015; Krukenberg et al., 2018; Kashyap and Holden, 2021 (for multihemes of Fe(III) reducing and ANME syntrophic archaea) | Custom gene database |
| Fe(II) oxidation | Multiheme cytochromes (PS51008 PROSITE Multiheme cytochrome *c* family proteins), totally, 20 entries of a custom database,  the Cyc2 neutrophilic iron oxidation pathway | ≥10  (at least, 1 membrane-associated and 1 qunone-reducing cytochrome of MtoABCD-like complex) | [MtoAB **OR** Cyc2] **AND** [MtoC **OR** CymA/Cbc **OR** Pcc complexes] | 20 **^5^** / 70 | Chakraborty et al., 2005; Emerson et al., 2007; Liu et al., 2012 (for MtoABCD proteins of anaerobic Fe(II) oxidizing bacteria),  McAllister et al., 2020 (for the Cyc2 neutrophilic iron oxidation pathway) | Custom gene database |
| Aerobic respiration | Oxidative phosphorylation – KEGG reference map00190, bacterial and archaeal entries | 80 | Catalytic subunits of respiratory complexes I & III (cytochrome *bc_1_* or alternative complex ACIII) **AND** [one of heme-copper terminal oxygen reductases (A-, B-, or C(*cbb_3_*)-type cytochrome *c* oxidases) **OR** a cytochrome *bd* complex] **AND** ATP synthase (F- or V/A- type) | 60 / 80 | KEGG database,  Golyshina et al., 2016 and references therein (for cytochrome *c* oxygen reductases and ACIII) | KEGG database  UniProt Knowledgebase |

^1^ Detailed lists of enzymes used to determine any of the mentioned carbon fixation pathways in the MAGs are provided in the **Supplementary Figure S5**. KEGG modules for the reductive glycine pathway and reverse oxidative TCA (roTCA) cycle are absent in the database, the necessary gene IDs were retrieved from Steffens et al., 2022 and Sánchez-Andrea et al., 2020.

^2^ “Custom databases” were manually compiled based on comprehensive analysis of publications related to the target metabolic pathway as by August 2024, query sequences for metagenomic analysis in custom databases were retrieved from several different public databases (UniProt, NCBI databases, etc.) or directly from scientific papers (if provided).

^3^ The description of the “CO to acetate pathway” and the genes necessary for its identification were taken from Schoelmerich & Müller, 2020.

^4^ Low identity percentage cutoff (30%) for CO-dehydrogenases was applied considering wide phylogenetic diversity of this group enzymes (Inoue et al., 2019).

^5^ Low identity percentage cutoff (20%) for multiheme cytochromes was applied considering wide phylogenetic diversity of this group enzymes, which is discussed in Gavrilov et al., 2021 and references therein.

^*^ The aerobic methane oxidation pathway and, in particular, *amoABC* genes were screened but were not detected in any of the MAGs analyzed.

**References for the Table S5.**

Steffens L, Pettinato E, Steiner TM, Eisenreich W, Berg IA. Tracking the Reversed Oxidative Tricarboxylic Acid Cycle in Bacteria. Bio Protoc. 2022. 12(6):e4364. doi: 10.21769/BioProtoc.4364.

Sánchez-Andrea I, Guedes IA, Hornung B, Boeren S, Lawson CE, Sousa DZ, Bar-Even A, Claassens NJ, Stams AJM. The reductive glycine pathway allows autotrophic growth of *Desulfovibrio desulfuricans*. Nat Commun. 2020. 11(1):5090. doi: 10.1038/s41467-020-18906-7.

Simon J, Klotz MG. Diversity and evolution of bioenergetic systems involved in microbial nitrogen compound transformations. Biochim Biophys Acta. 2013. 1827(2):114-35. doi: 10.1016/j.bbabio.2012.07.005.

Timmers PH, Welte CU, Koehorst JJ, Plugge CM, Jetten MS, Stams AJ. Reverse Methanogenesis and Respiration in Methanotrophic Archaea. Archaea. 2017. 2017:1654237. doi: 10.1155/2017/1654237.

Schoelmerich MC, Müller V. Energy-converting hydrogenases: the link between H_2_ metabolism and energy conservation. Cell Mol Life Sci. 2020. 77(8):1461-1481. doi: 10.1007/s00018-019-03329-5.

Inoue M, Nakamoto I, Omae K, Oguro T, Ogata H, Yoshida T, Sako Y. Structural and Phylogenetic Diversity of Anaerobic Carbon-Monoxide Dehydrogenases. Front Microbiol. 2019. 9:3353. doi: 10.3389/fmicb.2018.03353.

Kim YJ, Lee HS, Kim ES, Bae SS, Lim JK, Matsumi R, Lebedinsky AV, Sokolova TG, Kozhevnikova DA, Cha SS, Kim SJ, Kwon KK, Imanaka T, Atomi H, Bonch-Osmolovskaya EA, Lee JH, Kang SG. Formate-driven growth coupled with H_2_ production. Nature. 2010. 467(7313):352-5. doi: 10.1038/nature09375.

Greening C, Biswas A, Carere CR, Jackson CJ, Taylor MC, Stott MB, Cook GM, Morales SE. Genomic and metagenomic surveys of hydrogenase distribution indicate H_2_ is a widely utilized energy source for microbial growth and survival. ISME J. 2016. 10(3):761-77. doi: 10.1038/ismej.2015.153.

Chernyh NA, Neukirchen S, Frolov EN, Sousa FL, Miroshnichenko ML, Merkel AY, Pimenov NV, Sorokin DY, Ciordia S, Mena MC, Ferrer M, Golyshin PN, Lebedinsky AV, Cardoso Pereira IA, Bonch-Osmolovskaya EA. Dissimilatory sulfate reduction in the archaeon '*Candidatus* Vulcanisaeta moutnovskia' sheds light on the evolution of sulfur metabolism. Nat Microbiol. 2020. 5(11):1428-1438. doi: 10.1038/s41564-020-0776-z.

Sabuda MC, Brazelton WJ, Putman LI, McCollom TM, Hoehler TM, Kubo MDY, Cardace D, Schrenk MO. A dynamic microbial sulfur cycle in a serpentinizing continental ophiolite. Environ Microbiol. 2020. 22(6):2329-2345. doi: 10.1111/1462-2920.15006.

Gavrilov, S., Podosokorskaya, O., Alexeev, D., Merkel, A., Khomyakova, M., Muntyan, M., et al. 2017. Respiratory pathways reconstructed by multi-omics analysis in *Melioribacter roseus*, residing in a deep thermal aquifer of the west-Siberian Megabasin. Front. Microbiol. 8:1228. doi: 10.3389/fmicb.2017.01228.

Gavrilov, S. N., Zavarzina, D. G., Elizarov, I. M., Tikhonova, T. V., Dergousova, N. I., Popov, V. O., et al. 2021. Novel extracellular electron transfer channels in a gram-positive thermophilic bacterium. Front. Microbiol. 11:597818. doi: 10.3389/fmicb.2020.597818.

Mardanov, A. V., Slododkina, G. B., Slobodkin, A. I., Beletsky, A. V., Gavrilov, S. N., Kublanov, I. V., et al. (2015). The *Geoglobus acetivorans* genome: Fe(III) reduction, acetate utilization, autotrophic growth, and degradation of aromatic compounds in a hyperthermophilic archaeon. Appl. Environ. Microbiol. 81, 1003–1012. doi: 10.1128/AEM.02705-14.

Smith, J. A., Aklujkar, M., Risso, C., Leang, C., Giloteaux, L., and Holmes, D. E. (2015). Mechanisms involved in Fe(III) respiration by the hyperthermophilic archaeon *Ferroglobus placidus*. Appl. Environ. Microbiol. 81, 2735–2744. doi: 10.1128/AEM.04038-14.

Krukenberg, V., Riedel, D., Gruber-Vodicka, H. R., Buttigieg, P. L., Tegetmeyer, H. E., Boetius, A., et al. (2018). Gene expression and ultrastructure of meso- and thermophilic methanotrophic consortia. Environ. Microbiol. 20, 1651–1666. doi: 10.1111/1462-2920.14077.

Kashyap, S., and Holden, J. F. (2021). Microbe-mineral interaction and novel proteins for Iron oxide mineral reduction in the hyperthermophilic crenarchaeon *Pyrodictium delaneyi*. Appl. Environ. Microbiol. 87, 2320–2330. doi: 10.1128/AEM.02330-20.

Chakraborty, R., O’Connor, S. M., Chan, E., and Coates, J. D. (2005). Anaerobic degradation of benzene, toluene, ethylbenzene, and xylene compounds by *Dechloromonas strain* RCB. Appl. Environ. Microbiol. 71, 8649–8655. doi: 10.1128/AEM.71.12.8649-8655.2005.

Emerson, D., Rentz, J. A., Lilburn, T. G., Davis, R. E., Aldrich, H., Chan, C., et al. (2007). A novel lineage of proteobacteria involved in formation of marine Fe-oxidizing microbial mat communities. PLoS One 2:e667. doi: 10.1371/journal.pone.0000667.

Liu, J., Wang, Z., Belchik, S. M., Edwards, M. J., Liu, C., Kennedy, D. W., et al. (2012). Identification and characterization of MtoA: a decaheme c-type cytochrome of the neutrophilic Fe(II)-oxidizing bacterium *Sideroxydans lithotrophicus* ES-1. Front. Microbiol. 3:37. doi: 10.3389/fmicb.2012.00037.

McAllister SM, Vandzura R, Keffer JL, Polson SW, Chan CS. Aerobic and anaerobic iron oxidizers together drive denitrification and carbon cycling at marine iron-rich hydrothermal vents. ISME J. 2021. 15(5):1271-1286. doi: 10.1038/s41396-020-00849-y.

Golyshina OV, Kublanov IV, Tran H, Korzhenkov AA, Lünsdorf H, Nechitaylo TY, Gavrilov SN, Toshchakov SV, Golyshin PN. Biology of archaea from a novel family *Cuniculiplasmataceae* (*Thermoplasmata*) ubiquitous in hyperacidic environments. Sci Rep. 2016. 6:39034. doi: 10.1038/srep39034.

***Table S6.*** Estimated temperature of the groundwater within the aquifers calculated using different geothermometers. Wells are colored according to beta-diversity groups from Figure 2.

| **Well** | **70** | **46** | **49-E** | **71** | **9** | **75-bis** |
| --- | --- | --- | --- | --- | --- | --- |
| Well depth, m | 212 | 686 | 865 | 999 | 600 | 974 |
| Wellhead temperature, ^o^C | 19.7 | 35.6 | 30.2 | 40.5 | 21.9 | 20.2 |
| **Geothermometer** | **Estimated reservoir temperature (°C)** | | | | | |
| SiO_2_ (Fournier, 1977) | 12.7 | 62.0 | 64.6 | 68.5 | 21.2 | 53.8 |
| SiO_2_ (Giggenbach, 1983) | 15.0 | 67.1 | 69.8 | 74.0 | 23.9 | 58.3 |
| SiO_2_ (Verma & Santoyo, 1997) | 63.3 | 87.4 | 80.5 | 93.0 | 67.5 | 64.3 |
| K-Mg (Giggenbach, 1988) | 58.8 | 78.5 | 75.9 | 86.5 | 43.2 | 64.0 |
| Mg-Li (Kharaka & Mariner, 1989) | 31.5 | 49.7 | 67.2 | 69.4 | n.cor. | 42.6 |
| Na-Li (Kharaka & Mariner, 1989) | 69.3 | 66.3 | 84.2 | 84.4 | 55.0 | 128.9 |

***Table S7.*** Chemical and isotopic composition of the free gas discharges from YMWD wells.

Gas contents are given in %; δ^13^С in CO_2_ and CH_4_ - as ‰ V-PDB. Wells are colored according to beta-diversity groups from Figure 2.

| Well number | Units | **46** | **49-E** | **71** | **9** | **75-bis** |
| --- | --- | --- | --- | --- | --- | --- |
| Aquifer Abbreviation |  | К_2_s-m  **UC UC UC** | | | К_1_а-al_1_  **LC** | J_3_tt-K_1_v  **UJ** |
| Ar | % | 1.14 | 1.22 | ND | 0.77 | 0.06 |
| O_2_ |  | 0.23 | 0.52 | 0.53 | 19.72 | 0.05 |
| He |  | 0.07 | 0.25 | 0.02 | ND | 0.001 |
| H_2_ |  | 0.01 | 0.02 | 0.01 | ND | ND |
| N_2_ |  | 18.24 | 23.82 | 24.51 | 78.94 | 0.45 |
| CH_4_ |  | 3.12 | 11.85 | 8.92 | 0.02 | 0.004 |
| CO_2_ |  | 77.19 | 62.32 | 66.01 | 0.55 | 99.44 |
| δ^13^С, CH_4_ | ‰ V-PDB | -60.00 | -61.11 | -60.40 | ND | ND |
| δ^13^С, СО_2_ |  | -6.04 | -6.03 | -8.60 | -16.37 | -2,96 |
| N_2_/Ar |  | 16.00 | 21.98 | ND | 102.53 | 7.14 |
| N_2_/He |  | 260.57 | 107.28 | 1225.50 | ND | 409.09 |
| He/Ar |  | 0.06 | 0.20 | ND | ND | 0.02 |

ND – not determined

***Table S8.*** Grouping of wells by alfa-diversity calculations of corresponding microbial communities based on 688 phylotypes. Wells are colored according to beta-diversity grouping at Figure 2.

| Well No/ Abbreviation | Richness | Richness SD | Shannon.Index | Shannon.Index SD | Simpson.Index | Simpson.Index SD | Evenness | Evenness SD |
| --- | --- | --- | --- | --- | --- | --- | --- | --- |
| 70/ *RA (recharge area)* | 90.00 | 48.78 | 3.34 | 0.46 | 0.09 | 0.03 | 0.53 | 0.02 |
| 46/UC | 38.00 | 11.59 | 2.76 | 0.15 | 0.10 | 0.01 | 0.53 | 0.02 |
| 49-E/UC | 43.83 | 12.12 | 2.71 | 0.24 | 0.12 | 0.04 | 0.50 | 0.07 |
| 71/UC | 39.75 | 10.28 | 2.40 | 0.13 | 0.17 | 0.02 | 0.46 | 0.05 |
| 9/LC | 32.13 | 14.24 | 1.64 | 0.41 | 0.33 | 0.09 | 0.33 | 0.04 |
| 75-bis/UJ | 17.30 | 10.54 | 1.18 | 0.16 | 0.39 | 0.05 | 0.31 | 0.05 |
| **ANOVA F-value** | **24.75** |  | **103.73** |  | **75.656** |  | **62.07** |  |
| The analysis was performed using the software Rhea (Lagkouvardos et al., 2017).  SD – standard deviation. | | | | |  |  |  |  |
| **References for the Table S8:**  [Lagkouvardos I, Fischer S, Kumar N, Clavel T. (2017) Rhea: a transparent and modular R pipeline for microbial profiling based on 16S rRNA gene amplicons. PeerJ 5:e2836 https://doi.org/10.7717/peerj.2836](https://doi.org/10.7717/peerj.2836)  ***Table S9.*** Phylogenetic diversity of YMWB microbial communities at the genus level based on the results of high-throughput sequencing of 16S rRNA gene amplicons analyzed against the SILVA database, v.138, phyla names are corrected according to Oren & Garrity, 2022 (71). Presented as a separate MS Excel file. | | | | | | | | |

| ***Table S10***. Summarized results of the primary enrichments cultivation. | | | | | |
| --- | --- | --- | --- | --- | --- |
| **Target metabolic group** | UC aquifer | | | LC aquifer | UJ aquifer |
|  | **Wells** | | | | |
|  | **46** | **49-E** | **71** | **9** | **75-bis** |
| organotrophic sulfate reducers | no growth | low growth | *Spirochaetaceae* 61.04% (0.16%)  *Methanothermobacter* 19.62% (32.47%)  *Deferribacteraceae* 15.94% (0.05%)  *Anaerosomatales* 1.11% (3.48%) (**SAMN40263635**) | ND | no growth |
| lithotrophic sulfate reducers | no growth | *Pseudothermotoga* 17.35% (0.02%)  *Methanoculleus* 80.88% (0.01%) (**SAMN40263634**) | *Methanothermobacter* 98.55% (32.47%)  *Deferribacteraceae* 1.23% (0.05%) (**SAMN40263628**) | ND | no growth |
| organotrophic iron reducers | low growth* | *Anaerosomatales* 6.53% (8.49%)  *Spirochaetaceae* 92.81% (0.16%)  (**SAMN40263626**) | *Deferribacteraceae* 53.53% (0.05%)  *Anaerosomatales* 36.10% (3.48%)  *Spirochaetaceae* 10.05% (0.16%) *(***SAMN40263625***)* | ND | no growth |
| lithotrophic iron reducers | low growth |  | *Rhodocyclaceae* 99.31% (0.01%) (**SAMN40263627**) | ND | no growth |
| anaerobic iron oxidizers | low growth |  | *Deferribacteraceae* 43.08% (0.05%)  *Spirochaetaceae* 14.35% (0.16%)  *Hydrogenophilus* 24.86% (0.01%)  *Rhodocyclaceae* 4.85% (0.1%)  *Anaerosomatales* 10.77% (3.48%) (**SAMN40263630**) | ND | no growth |
| hydrogenotrophic methanogens | no growth | *Methanothermobacter* 57% (8.97%)  *Thermodesulfovibrio* 41% (2.9%)  *Pseudothermotoga* 2% (0.02%)  (**SAMN43543792**) | *Methanothermobacter* 98.55% (32.47%) (**SAMN40263631**) | ND | low growth |
| organotrophic Archaea | no growth | *Methanothermobacter* 39.51% (8.97%)  *Methanoculleus* 57.89% (0.01%) (**SAMN40263638**) | *Methanothermobacter* 98.12% (32.47%) (**SAMN40263639**) |  | low growth |
|  |  |  |  | ND |  |
| acetate oxidizers | no growth | low growth | *Desulfosoma* 90.54% (0.01%)  *Methanothermobacter* 7.51% (32.47%) (**SAMN40263636**) |  | no growth |
|  |  |  |  | ND |  |
| organotrophs | *Proteiniphilum* 99.36% (0.64%) (**SAMN40263633**) | *Pseudothermotoga* 98.82% (0.02%) (**SAMN40263632**) | *Thermogutta* 72.66% (0.01%)  *Candidatus* Caldatribacterium 25.09% (0.01%)  *Methanothermobacter* 1.55% (32.47%) (**SAMN40263637**) | Data published previously** | no growth |
|  |  |  |  |  |  |
| The relative abundance of the detected taxa is given for the enrichments and for the corresponding water samples (in parentheses) used to obtain these enrichments. | | | | | |
| The cells in the table are colored according to the incubation time required for the enrichments to reach the stationary phase as seen by microscopy. The color code is as follows. | | | | | |
| Time of incubation: | 4-5 days | two weeks | four weeks | no growth was observed within 4 weeks of incubation | |
|  |  |  |  |  |  |
| ND -no data  *The "low growth" sign indicates those cultures that did not exceed a density of approximately 10^5^ cells per mL by the time they reached the stationary growth phase. The low cell density did not allow sufficient DNA to be isolated for 16S rRNA profiling of these cultures.  **Podosokorskaya OA, Elcheninov AG, Gavrilov SN, Petrova NF, Klyukina AA, et al. New representatives of the class *Ignavibacteria* inhabiting subsurface aquifers of Yessentuki mineral water deposit. Water 2023; 15:3451. | | | | | |
